# Construction and Validation of Plasma Protein‐Based Musculoskeletal Biological Age and Genetic and Environmental Risk Profiles

**DOI:** 10.1111/acel.70636

**Published:** 2026-07-25

**Authors:** Yangdan Zhong, Maoyao Xia, Xunying Zhao, Yang Qu, Bowen Lei, Sirui Zhen, Tao Han, Rong Xiang, Jinyu Xiao, Xin Song, Xiaofeng Ma, Bin Yang, Di Zhang, Jinyu Zhou, Zilan Chen, Yuqi Pang, Ye Ju, Ting Liu, Zihao Li, Lu Long, Tao Zhang, Jiayuan Li, Mengyu Fan, Zuyun Liu, Xia Jiang

**Affiliations:** ^1^ Department of Nutrition and Food Hygiene, West China School of Public Health and West China Fourth Hospital Sichuan University Chengdu China; ^2^ Department of Epidemiology and Health Statistics, West China School of Public Health and West China Fourth Hospital Sichuan University Chengdu China; ^3^ Second Affiliated Hospital, School of Public Health, Zhejiang Key Laboratory of Intelligent Preventive Medicine Zhejiang University School of Medicine Hangzhou China; ^4^ Department of Clinical Neuroscience Karolinska Institutet Stockholm Sweden

**Keywords:** aging clock, drug targets, GWAS, musculoskeletal aging, musculoskeletal protein pool

## Abstract

The musculoskeletal system is key to aging. Nonetheless, existing protein‐based musculoskeletal aging clocks have limited predictive performance and fail to characterize genetic or environmental determinants. We first constructed a musculoskeletal protein pool by integrating transcriptomic enrichment, protein functional annotation, and literature expertise. Two musculoskeletal aging clocks—MSKAge and MSKAgeMort—were then developed using proteomic data of 21,070 UK Biobank participants. Acceleration metric (MSKAgeAccel/MSKAgeMortAccel) quantified deviations of biological age from chronological age, with positive values indicating accelerated musculoskeletal aging. Associations of MSKAgeAccel/MSKAgeMortAccel with mortality, musculoskeletal disorders, and age‐related diseases were assessed using Cox proportional hazards models. Environmental and genetic determinants were evaluated by linear regression and genome‐wide association analyses, respectively. Drug repurposing was used to identify potential targets of musculoskeletal diseases. A total of 39 musculoskeletal‐related proteins constituted the pool. Leveraging these proteins, MSKAge (*r*_female_ = 0.62; *r*_male_ = 0.56) and MSKAgeMort (*r*_female_ = 0.93; *r*_male_ = 0.88) were constructed. Both MSKAgeAccel and (in particular) MSKAgeMortAccel predicted mortality, musculoskeletal diseases, and age‐related diseases effectively. MSKAgeMortAccel showed significant associations with musculoskeletal disorders, including osteoarthritis, rheumatoid arthritis, gout, and low back pain. MSKAgeMortAccel was influenced by environmental pollutants, psychological factors, and unhealthy lifestyles. Genetic analyses revealed 13 variants and 71 genes enriched in epigenetic regulation and extracellular environment‐receptor interaction. Zinc was identified as a candidate musculoskeletal drug. We established a reliable musculoskeletal aging clock and elucidated genetic and environmental determinants. This framework enables stratification of individuals at risk of accelerated musculoskeletal aging, paving the way for anti‐aging interventions.

## Graphical abstract

We developed plasma protein‐based musculoskeletal aging clocks, MSKAge and MSKAgeMort, using UK Biobank proteomic data, and showed that MSKAgeAccel and MSKAgeMortAccel (in particular) predicted mortality, musculoskeletal disorders, and age‐related diseases effectively. MSKAgeMortAccel further revealed environmental and genetic profiles, with zinc highlighted as a candidate intervention. Created with assistance from ChatGPT image generation tools and finalized by the authors.

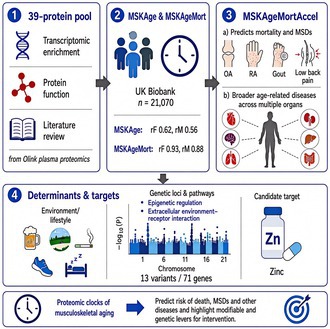

## 1 Introduction

Aging is a fundamental biological process characterized by progressive physiological decline and increased disease susceptibility. Notably, the rate of functional decline varies among individuals with the same chronological age (CA) (Hamczyk et al. 2020; Kennedy et al. 2014). To quantify this heterogeneity, biological age clocks have been developed leveraging clinical indicators (Levine 2013) or multi‐omics data (Rutledge et al. 2022), demonstrating superior performance over CA in predicting morbidity and mortality. However, these clocks mainly reflect systemic aging and overlook organ‐specific aging trajectories (Rutledge et al. 2022). Developing organ‐specific aging clocks is therefore critical, as they can capture tissue‐specific changes that may be masked by the systemic assessments. This precision enables more accurate risk stratification and paves the way for targeted interventions.

As a direct physical manifestation of aging, the musculoskeletal system undergoes progressive deterioration characterized by muscle mass loss, bone density decline, and joint degeneration, which can lead to musculoskeletal disorders (MSDs), disability, and death that affect billions of individuals worldwide (GBD 2017 Disease and Injury Incidence and Prevalence Collaborators 2018). MSDs often occur sequentially or simultaneously and exacerbate each other, severely compromising mobility, independence, and quality of life, imposing healthcare and socioeconomic burdens. Thus, a dedicated musculoskeletal aging clock is needed to enable early risk detection, timely interventions, and reduction of disease burden.

Several musculoskeletal aging clocks have been developed, including phenotypic (Tian et al. 2023), epigenetic (Green et al. 2025), and proteomic (Goeminne et al. 2025) clocks, yet limitations remain. Phenotypic clocks rely on crude functional measurements such as grip strength, which usually fail to capture early‐stage dysfunction and subclinical aging. Epigenetic clocks offer biological insight but require invasive tissue sampling, limiting clinical scalability. Proteins, as downstream functional products of gene expression and regulators of biological pathways, provide valuable aging trajectories of different organs (Goeminne et al. 2025), achieving a balance among functional relevance, organ specificity, and clinical accessibility, and thus act as the preferred choice for constructing organ‐specific aging clocks. Leveraging plasma proteomics from over 50,000 UK Biobank (UKB) participants and other cohorts, Goeminne and colleagues developed musculoskeletal aging clocks (MuscAge) through a data‐driven approach integrating tissue‐specific transcription patterns from the Genotype‐Tissue Expression (GTEx) project to identify organ‐enriched proteins (Goeminne et al. 2025). However, several critical gaps remain: first, the predictive performance was poor, with the best‐performing model reaching only marginal correlation (*r* = 0.26–0.28); second, the selection of merely skeletal muscle ignored other important tissues such as bone; third, the protein pool was narrow with candidate proteins screened solely based on organ‐specific enrichment in gene expression levels without sufficient functional annotation or literature expertise, limiting biological interpretability; finally, the determinants and actionable interventional targets of musculoskeletal aging remained uncharacterized.

**FIGURE 1:**
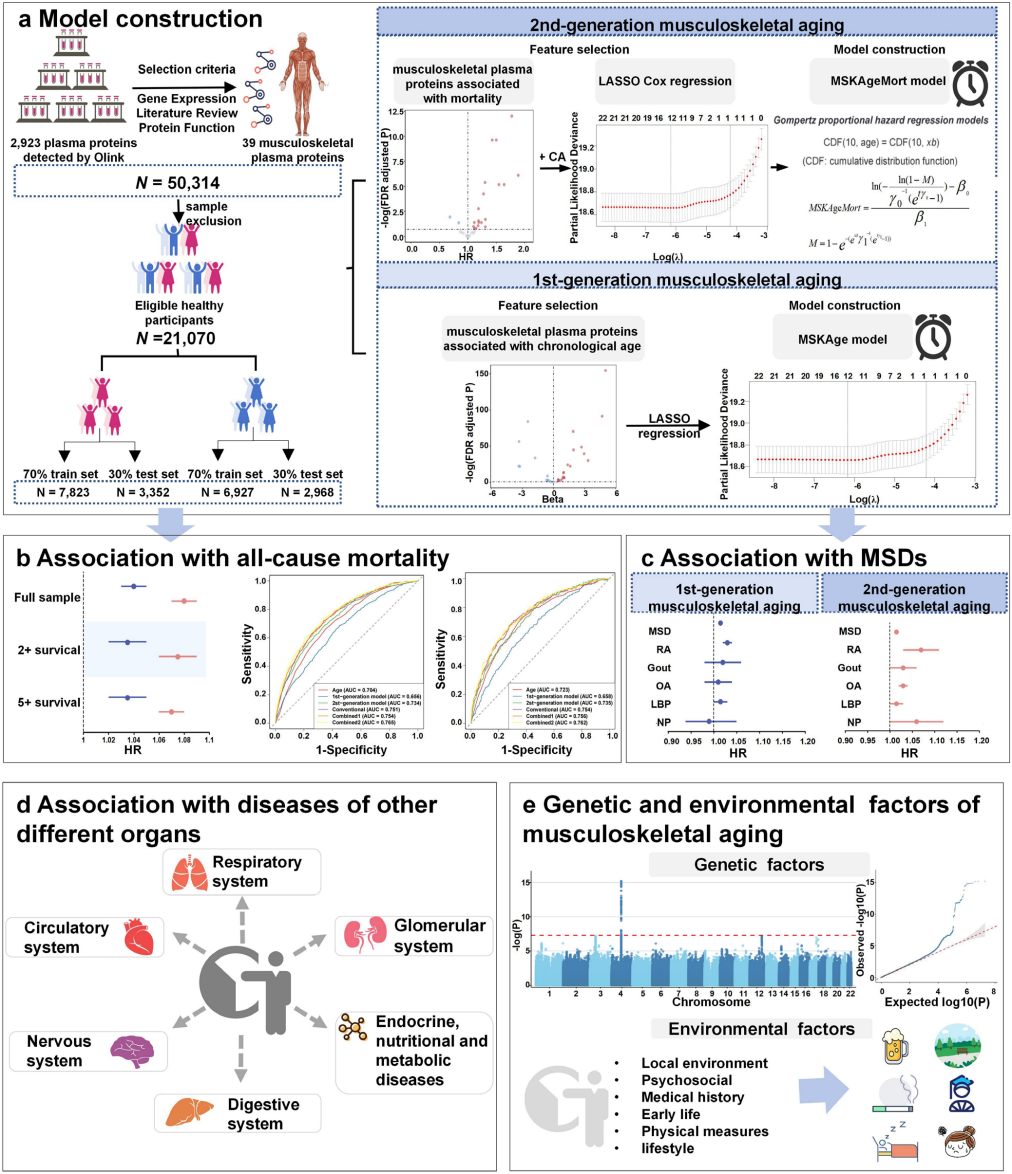
Overview of study design. (a) 39 musculoskeletal‐related proteins make up the musculoskeletal protein pool. Musculoskeletal aging models were then trained on 70% of 21,070 UK Biobank participants and tested on the remaining 30%. For the 2nd‐generation musculoskeletal aging model, 21 female‐specific and 25 male‐specific musculoskeletal plasma proteins associated with all‐cause mortality, along with CA were screened by LASSO‐penalized Cox regression. Then MSKAgeMort was developed using Gompertz proportional hazards regression models. For 1st‐generation musculoskeletal aging model, 30 female‐specific and 30 male‐specific musculoskeletal plasma proteins associated with CA were used to develop MSKAge through LASSO regression. (b) Association analyses of MSKAgeMortAccel/MSKAgeAccel with all‐cause mortality. Receiver operating characteristic curves were used to evaluate the predictive power of MSKAgeMort/MSKAge for 10‐year mortality. (c) Association analyses of MSKAgeMortAccel/MSKAgeAccel with MSDs. (d) Association analyses of MSKAgeMortAccel/MSKAgeAccel with age‐related diseases of other different organs, including endocrine, nutritional and metabolic diseases (e.g., type 2 diabetes, hyperlipidemia), circulatory system (e.g., cardiovascular disease, hypertension, cerebrovascular diseases), digestive system (e.g., liver diseases, cirrhosis), respiratory system (e.g., chronic obstructive pulmonary disease), renal system (e.g., chronic kidney disease), nervous system (e.g., Alzheimer's disease, depression). (e) Genetic and environmental factors for MSKAgeMortAccel. *p < 0.05, **p < 0.01, ***p < 0.001. LASSO, least absolute shrinkage and selection operator; LBP, low back pain; MSD, musculoskeletal disorder; MSKAgeAccel, MSKAge acceleration; MSKAgeMortAccel, MSKAgeMort acceleration; NP, neck pain; OA, osteoarthritis; RA, rheumatoid arthritis.

In the study, we first constructed a musculoskeletal protein pool covering both muscle‐ and bone‐enriched proteins by integrating transcriptomic enrichment, protein functional annotation, and literature expertise. We then constructed a musculoskeletal aging clock based on mortality (MSKAgeMort), known as the 2nd‐generation aging model, complemented with a CA‐based clock (MSKAge), known as the 1st‐generation aging model. We further quantified musculoskeletal aging acceleration through linear regression residuals to (1) elucidate their relationships with all‐cause mortality, MSDs, and age‐related diseases; and (2) explore genetic and environmental determinants of musculoskeletal aging (Figure 1).

## 2 Results

### 2.1 Population Characteristics

The study design is shown in Figure S1. A total of 21,070 eligible participants free from pre‐existing conditions affecting musculoskeletal health (55.08 ± 8.31 years, 53.0% female, 92.3% white ethnicity) were included. Given sex‐specific differences in sociodemographic characteristics and lifestyle factors, sex‐stratified musculoskeletal aging clocks were developed (Table S1).

### 2.2 Candidate Musculoskeletal‐Related Proteins

Musculoskeletal aging clock construction relies on musculoskeletal‐related proteins involved in the formation and functional maintenance of skeletal muscle or bone. Our musculoskeletal protein pool was assembled from multiple sources. First, we incorporated 20 muscle‐enriched proteins previously identified in UKB proteomics using GTEx (Goeminne et al. 2025). To this core set, we added six well‐established muscle‐enriched proteins identified through functional annotation and literature review. For bone‐specific proteins, the absence of bone tissue data in GTEx precluded a similar genomic approach; therefore, we identified 13 bone‐enriched proteins solely through functional annotation and literature review. In total, this curated pool comprises 39 candidate proteins (Table S2).

### 2.3 Plasma Proteome Supports Musculoskeletal Aging Clock Construction

A total of 21,070 participants were randomly split into training (70%, *N*_female_ = 7823, and *N*_Male_ = 6927) and testing (30%, *N*_female_ = 3352, *N*_Male_ = 2968) sets to develop musculoskeletal aging clocks (Figure S1). Within each sex, baseline characteristics were well balanced between training and testing sets (Tables S3 and S4).

**FIGURE 2:**
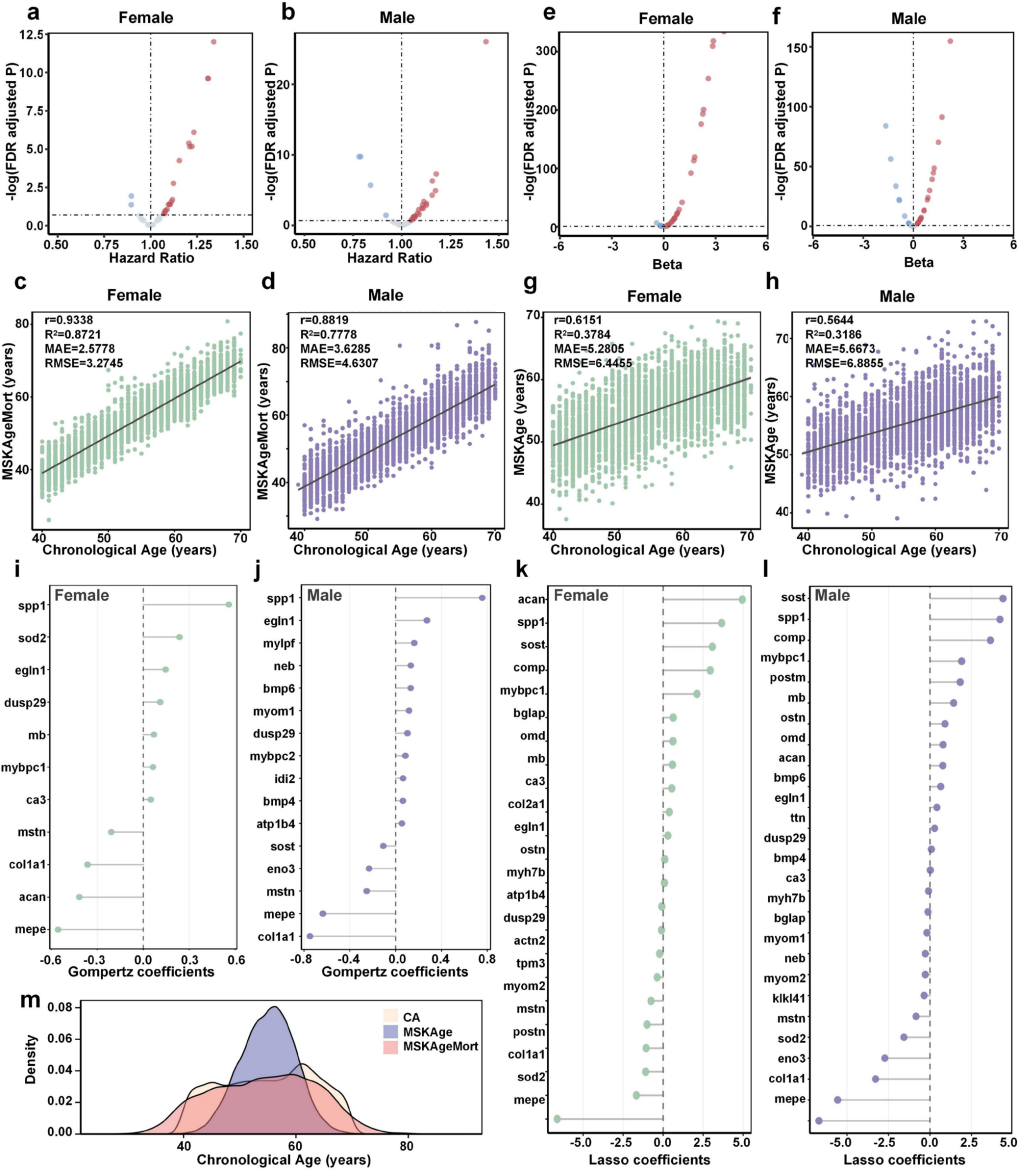
Development and performance of MSKAgeMort and MSKAge. (a, b) Volcano plots illustrating the association between musculoskeletal‐related proteins and CA: (a) female and (b) male. Red points indicate positive correlations, blue points indicate negative correlations, and gray points indicate no significant associations. The threshold is set at *p*_FDR_ < 0.2. (c, d) Scatter plots illustrating the performance of the MSKAgeMort model against CA in the testing set: (c) female and (d) male. Each scatter indicates a single participant. Pearson correlation coefficient (r), coefficient of determination (*R* ^2^), Mean Absolute Error (MAE), and Root Mean Squared Error (RMSE) are shown. The black line represents the linear regression fit. (e, f) Volcano plots illustrating the association between musculoskeletal‐related proteins and mortality: (e) female and (f) male, with more information refer to (a, b). (g, h) Scatter plots illustrating the performance of the MSKAge model against CA in the testing set: (g) female and (h) male, with more information refer to (c, d). (i, j) Corresponding Gompertz coefficients of the variable components of the MSKAgeMort model: (i) female and (j) male. (k, l) Lasso coefficients of the variable components of the MSKAge model: (k) female (l) male. (m) Density plots of CA (yellow), MSKAge (purple) and MSKAgeMort (red) distribution. CA, chronological age; MSKAgeAccel, MSKAge acceleration; MSKAgeMortAccel, MSKAgeMort acceleration.

To ensure the robustness of conclusions, both 1st‐ and 2nd‐generation clocks were developed. We analyzed data using the 2nd‐generation musculoskeletal aging model for primary analysis and the 1st‐generation musculoskeletal aging model for sensitivity validation. For the 2nd‐generation musculoskeletal aging model, 1569 deaths occurred during a median follow‐up of 13.82 years. A total of 21 female‐specific and 25 male‐specific musculoskeletal proteins (17 overlapping) significantly correlated with all‐cause mortality (*p*_FDR_ < 0.2, Figures 2a,b and S2a, Table S5). To refine feature selection, we used LASSO‐penalized Cox regression with 10‐fold cross‐validation on the training set, regressing all‐cause mortality risk against CA and the 21/25 proteins, selecting 11 proteins in females and 16 proteins in males, along with CA (Table S6). Using these mortality‐associated proteins, MSKAgeMort was developed using methods proposed by Levine et al. (2018) in the training set. Model performance was evaluated in the testing set and showed strong correlations with CA (*r*_female_ = 0.93, MAE_female_ = 2.58 years; *r*_male_ = 0.88, MAE_male_ = 3.63 years, Figure 2c,d).

For the 1st‐generation musculoskeletal aging model, we identified 30 female‐specific and 30 male‐specific musculoskeletal proteins (28 overlapping) associated with CA (*p*_FDR_ < 0.2, Figures 2e,f and S2b, Table S7). Then LASSO regression with 10‐fold cross‐validation in the training set yielded 23 and 26 non‐zero coefficients for females and males. MSKAge showed moderate performance in the testing set (*r*_female_ = 0.62, MAE_female_ = 6.28 years; *r*_male_ = 0.56, MAE_male_ = 5.67 years, Figure 2g,h), surpassing MuscAge constructed by Goeminne et al. (2025), but inferior to MSKAgeMort.

Additional parameters were shown in Figure 2i–l and Tables S8 and S9. MSKAgeMort demonstrated superior alignment with CA compared to MSKAge (Figure 2m), with a wider range of age estimates (24.93–89.91 vs. 33.81–75.08 years). While both models showed similar median values (54.71 years), MSKAgeMort exhibited greater dispersion (standard deviation [SD] = 9.31, interquartile range [IQR] = 46.93–61.46) than MSKAge (SD = 4.81, IQR = 51.79–58.42), reflecting its capacity to capture a full spectrum of aging. We quantified acceleration of both MSKAge and MSKAgeMort, with distributions visualized in Figure S3.

### 2.4 Functional Convergence and Divergence in Musculoskeletal‐Related Proteins

To investigate the functional characteristics of the identified proteins, we conducted systematic enrichment analyses. Both CA‐ and mortality‐associated proteins showed functional convergence across sexes. Gene Ontology (GO) enrichment analysis showed that the 28 shared CA‐associated proteins were enriched in actin binding, sarcomere organization, and extracellular matrix (ECM)‐receptor interaction, whereas 17 shared mortality‐associated proteins were enriched in muscle structure and contraction, ECM integrity, and skeletal development signaling (Figure S4a). Kyoto Encyclopedia of Genes and Genomes (KEGG) analyses further highlighted ECM‐receptor interaction and Cytoskeleton in muscle cells pathways (Figure S4b). Beyond the shared biological foundation, sex‐specific differences were observed. Male‐specific mortality‐associated proteins were enriched in sarcomere organization and myosin filament integrity, suggesting a stronger contribution of contractile apparatus impairment. Conversely, female‐specific mortality‐associated proteins were associated with bone development, ossification, and metal ion binding (Figure S4a), indicating greater involvement of ECM homeostasis and oxidative stress. Similar patterns were found for CA‐associated proteins, suggesting structural and mechanical decay may dominate in males, whereas ECM homeostasis and remodeling may play a greater role in females.

### 2.5 Musculoskeletal Aging Accelerations Reflect Systemic Aging

**FIGURE 3:**
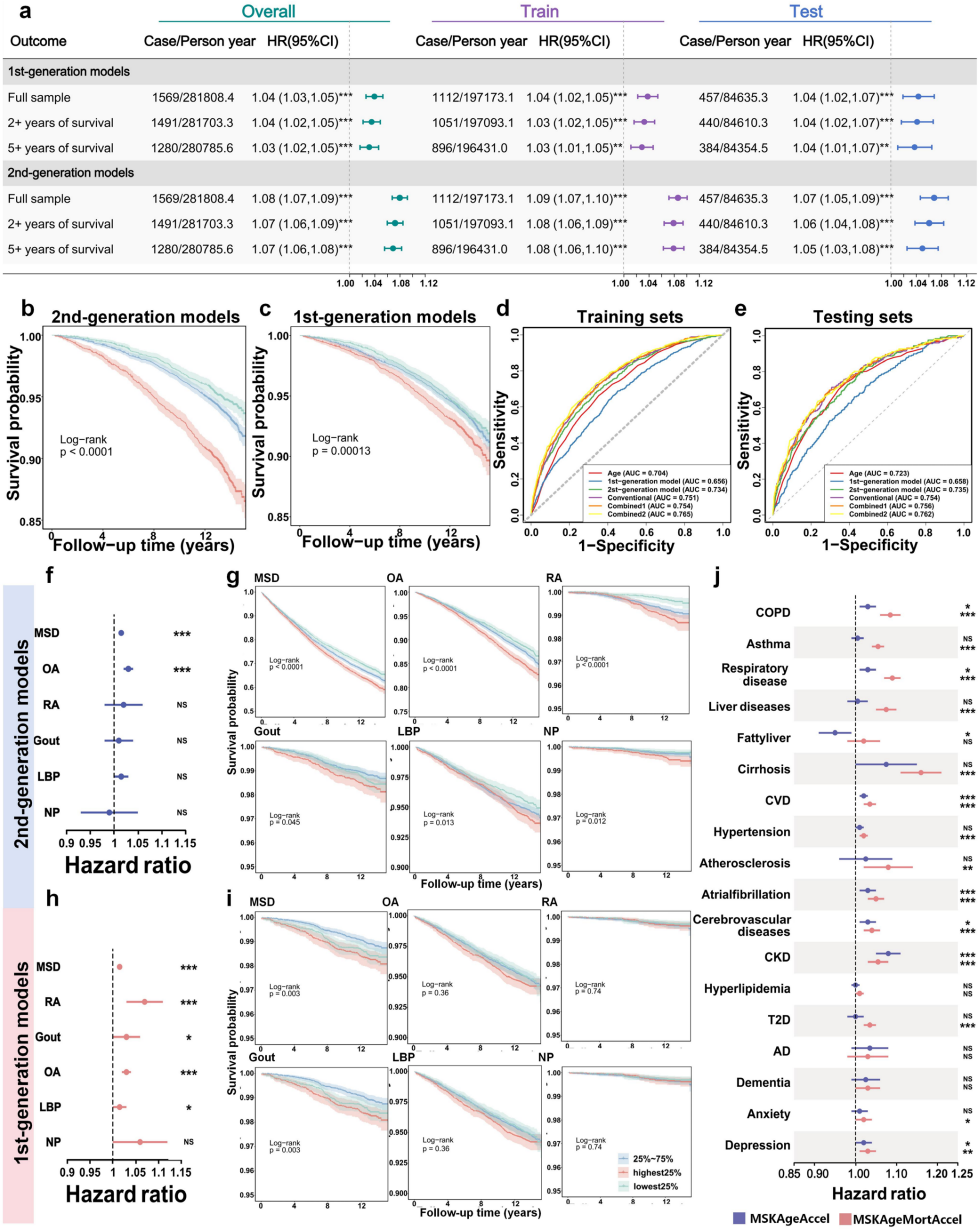
MSKAgeMortAccel and MSKAgeAccel in relation to all‐cause mortality, musculoskeletal disorders and age‐related diseases. (a) Forest plots showing HR with 95% CIs for the associations of MSKAgeMortAccel and MSKAgeAccel with all‐cause mortality in the overall (green), training (purple) and testing (blue) populations. We also repeated the analysis in those with 2+ and 5+ years of survival. (b, c) Kaplan–Meier survival plots of all‐cause mortality according to quartiles of the musculoskeletal aging acceleration: (b) MSKAgeMortAccel and (c) MSKAgeAccel. Red, blue, and green lines represent the top, combined middle two, and bottom quartiles, respectively, each with 95% confidence intervals. (d, e) Receiver operating characteristic curves for 10‐year all‐cause mortality in: (d) the training sets and (e) the testing sets. The six models compared include: CA‐only, MSKAge‐only/MSKAgeMort‐only, conventional risk factors (including CA, sex, ethnicity, TDI, education level, physical activity, BMI, smoking, and alcohol consumption status), and combined (conventional + MSKAge/MSKAgeMort) models. (f) Forest plots showing HR with 95% CIs for the associations of MSKAgeMortAccel with MSDs in the overall population. (g) Kaplan–Meier survival plots of MSDs according to quartiles of the MSKAgeMortAccel, with more information refer to (b, c). (h) Forest plots showing HR with 95% CIs for the associations of MSKAgeAccel with MSDs in the overall population. (i) Kaplan–Meier survival plots of MSDs according to quartiles of the MSKAgeAccel. (j) Forest plots showing HR with 95% CIs for the associations of MSKAgeMortAccel (read) and MSKAgeAccel (blue) with age‐related diseases. All Cox models adjusted for chronological age, sex, ethnicity, Townsend deprivation index, education level, physical activity, body mass index, smoking status, alcohol status, and sleep duration. *p < 0.05, **p < 0.01, ***p < 0.001. AD, Alzheimer's disease; CI, confidence interval; CKD, chronic kidney disease; COPD, chronic obstructive pulmonary disease; CVD, cardiovascular disease; HR: hazard ratio; LBP, low back pain; MSD, musculoskeletal disorder; MSKAgeAccel, MSKAge acceleration; MSKAgeMortAccel, MSKAgeMort acceleration; NP, neck pain; OA, osteoarthritis; RA, rheumatoid arthritis; T2D, type 2 diabetes.

Figure 3a shows the relationship of MSKAgeMortAccel and MSKAgeAccel with all‐cause mortality. After adjusting for CA, sex, ethnicity, Townsend deprivation index (TDI), education level, physical activity, body mass index (BMI), smoking status, alcohol status, and sleep duration, each 1‐year increase in MSKAgeMortAccel corresponded to an 8% increased risk of all‐cause mortality (HR = 1.08, 95% CI = 1.07–1.09). The finding remained robust after excluding participants who died within 2 or 5 years of follow‐up, restricting analyses to training/testing sets, and stratifying by sex. Similar but attenuated effects were observed for MSKAgeAccel, with each 1‐year increase associated with a 4% increased risk of mortality (HR = 1.04, 95% CI = 1.03–1.05) (Figure 3a; Table S10).

Kaplan–Meier (KM) survival curves demonstrated clear mortality risk stratification. Individuals in the highest MSKAgeMortAccel quartile (Q4) exhibited a greater risk of mortality than those in the lowest quartile (Q1) (Figure 3b). MSKAgeAccel showed comparable but less stark stratification (Figure 3c).

In addition to these crucial associations, MSKAgeMortAccel significantly improved the prediction of 10‐year mortality beyond CA. The MSKAgeMort model substantially outperformed CA in the Receiver Operating Characteristic (ROC) analysis. When integrated into a conventional risk factor model, mortality prediction was further enhanced, where robustness was confirmed in both training (AUC increase: +0.03) and testing (AUC increase: +0.01) sets (Figure 3d,e). In contrast, MSKAge offered minimal predictive utility.

### 2.6 Musculoskeletal Aging Accelerations Predict MSDs

Given that MSDs represent phenotypes of accelerated musculoskeletal aging, we next assessed the ability of our clocks to predict their incidence. After full adjustment, MSKAgeMortAccel showed significant associations with several well‐known high‐burden musculoskeletal conditions, including osteoarthritis (OA, HR = 1.03, 95% CI = 1.02–1.04), rheumatoid arthritis (RA, HR = 1.07, 95% CI = 1.03–1.11), gout (HR = 1.03, 95% CI = 1.00–1.06), and low back pain (LBP, HR = 1.02, 95% CI = 1.00–1.03), with neck pain (NP, HR = 1.06, 95% CI = 1.00–1.12) as an exception, potentially due to its limited sample size. Overall, each 1‐year increase in MSKAgeMortAccel conferred a 2% increased MSD risk (HR = 1.02, 95% CI = 1.01–1.02) (Figure 3f, Table S11). These associations remained consistent across sexes and training/testing sets, albeit with a few non‐significant findings that may reflect limited power (Table S12). When categorized into quartiles, KM curves revealed a dose–response relationship, with individuals in Q4 experiencing greater cumulative incidence of MSDs compared to those in Q1 (Figure 3g, Table S11). Consistently, MSKAgeAccel showed weaker but still significant associations with MSDs (Figure 3h,i, Table S11).

### 2.7 Musculoskeletal Aging Accelerations Provide Insights Into Other Age‐Related Diseases

Beyond MSDs, musculoskeletal aging accelerations were associated with multiple age‐related diseases across respiratory, digestive, circulatory, renal, endocrine, nutritional and metabolic, and nervous systems. After full adjustment, the top 10 diseases with the most significant elevated risk associated with MSKAgeMortAccel were cirrhosis (HR = 1.17, 95% CI = 1.12–1.22), chronic obstructive pulmonary disease (COPD, HR = 1.09, 95% CI = 1.07–1.10), respiratory diseases (HR = 1.09, 95% CI = 1.07–1.10), liver disease (HR = 1.07, 95% CI = 1.05–1.09), atherosclerosis (HR = 1.06, 95% CI = 1.02–1.11), chronic kidney disease (CKD, HR = 1.05, 95% CI = 1.03–1.07), fatty liver (HR = 1.04, 95% CI = 1.01–1.07), atrial fibrillation (HR = 1.04, 95% CI = 1.03–1.06), cerebrovascular diseases (HR = 1.04, 95% CI = 1.03–1.06), and Alzheimer's disease (AD, HR = 1.04, 95% CI = 1.00–1.08). For other diseases, please read Figure 3j and Table S13.

MSKAgeAccel showed similar associations with diseases of the renal, circulatory, nervous, respiratory, and endocrine, nutritional and metabolic systems, with CKD exhibiting the strongest association, followed by atrial fibrillation, cerebrovascular diseases, AD, depression, respiratory diseases, COPD, cardiovascular disease (CVD), and hypertension (Figure 3j, Table S13). While retaining modest predictive capacity, MSKAgeAccel showed inferior performance to MSKAgeMortAccel, particularly in the digestive system. All these association patterns were broadly consistent across sexes and training/testing sets (Table S14).

### 2.8 Robustness of Musculoskeletal Aging Clocks

To evaluate the influence of reverse causality and undiagnosed preclinical conditions, we conducted a sensitivity analysis by excluding individuals who died or developed diseases affecting the musculoskeletal system within 2 years after baseline (*N* = 19,767). The selected proteins remained highly consistent between primary and sensitivity analyses, with Pearson correlation of effect sizes exceeding 0.98 across sexes (Figure S5a–h). The retrained models also showed substantial structural stability (coefficient correlation *r* = 0.835–0.992, Figure S5i–p). Moreover, associations of musculoskeletal aging accelerations with mortality and MSDs remained consistent with the primary analyses (Figure S5q–t, Tables S15–S19). Together, our musculoskeletal aging clocks are unlikely to be influenced by preclinical diseases present at baseline.

### 2.9 Comparisons With Existing Proteomic Musculoskeletal Aging Clocks

**FIGURE 4:**
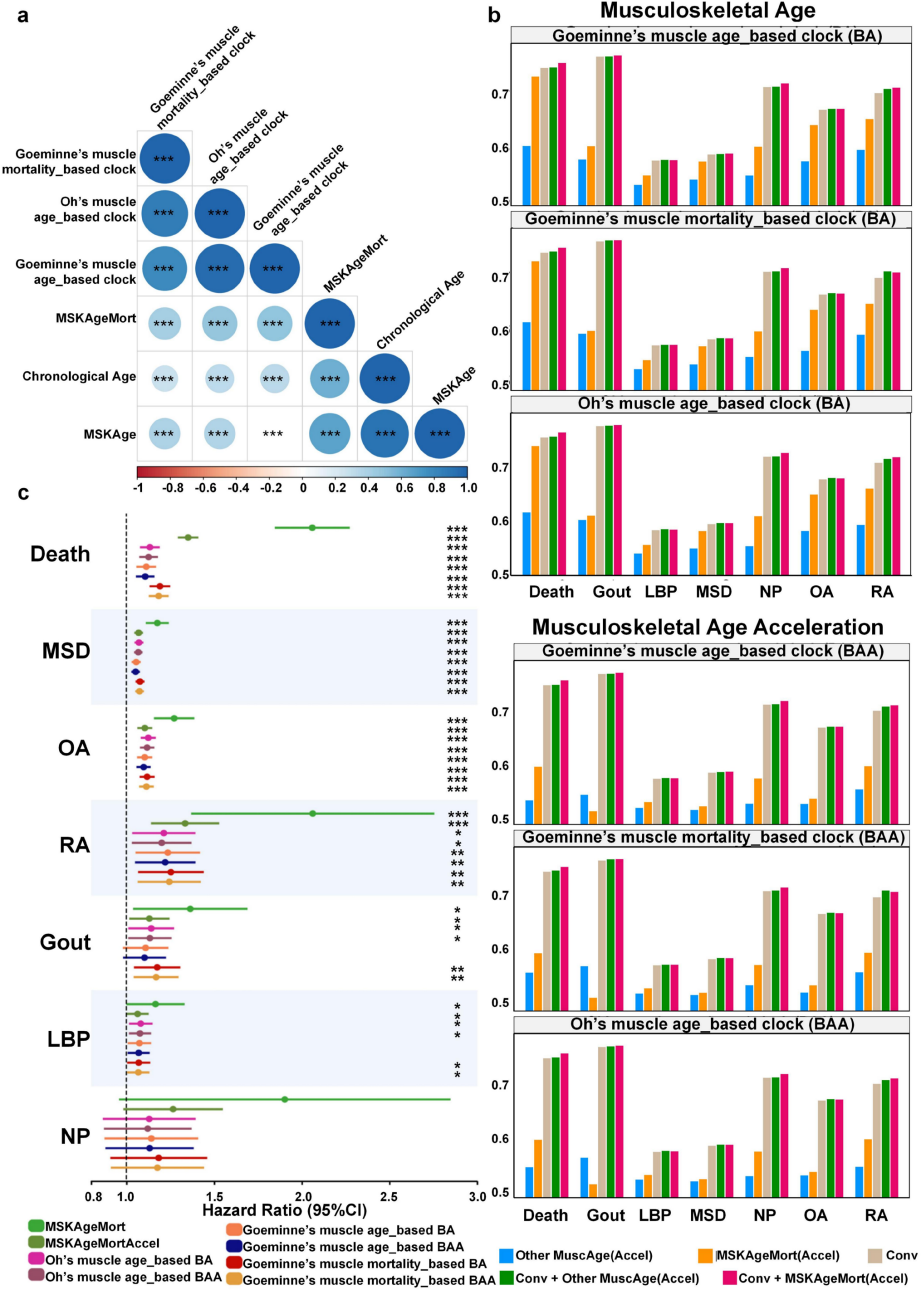
Comparison of MSKAgeMort with published proteomic musculoskeletal aging clocks. (a) Spearman correlation matrix among chronological age, MSKAge, MSKAgeMort, and three previously published proteomic musculoskeletal aging clocks. Circle size and color indicate the magnitude and direction of the correlation; asterisks denote statistical significance. (b) Comparative predictive performance of BA and BAA measures across seven clinical outcomes. Model performance was evaluated using the C‐index for each aging clock alone, conventional clinical models, and models combining conventional covariates with each aging measure. (c) Cox proportional hazards regression analyses comparing the associations of standardized BA and BAA measures with all‐cause death and MSDs. Points represent hazard ratios per SD increase, and horizontal lines represent 95% confidence intervals. Conv indicates the conventional clinical model adjusted for chronological age, sex, ethnicity, body mass index, Townsend deprivation index, smoking status, alcohol intake, education level, and physical activity. BA, biological age; BAA, biological age acceleration; CI, confidence interval; HR, hazard ratio; LBP, low back pain; MSD, musculoskeletal disorders; NP, neck pain; OA, osteoarthritis; RA, rheumatoid arthritis.

Given the superior performance of the 2nd‐generation musculoskeletal aging model, we compared MSKAgeMort with three published proteomic musculoskeletal aging clocks: a CA‐based clock from Oh et al. (2025) and two clocks trained on CA or mortality from Goeminne et al. (2025). MSKAgeMort was positively correlated with all three published clocks (Spearman's *r* = 0.38–0.50, *p* < 0.001, Figure 4a). For all‐cause mortality and most MSDs, MSKAgeMort showed better stand‐alone predictive performance than existing clocks. When added into a comprehensive clinical model, MSKAgeMort also achieved higher C‐indices and lower AIC values for most outcomes (Figure 4b, Table S20). Such comparable patterns were observed for MSKAgeMortAccel. Cox regression using standardized metrics further showed that MSKAgeMort generally had larger HRs per SD increase than the existing clocks across most outcomes (Figure 4c, Table S21). Similarly, MSKAgeMortAccel showed stronger associations with all‐cause mortality and RA, while associations for OA, LBP, and MSD were broadly comparable across existing clocks. Notably, biological age acceleration measures from all clocks showed limited associations with NP.

### 2.10 Environmental Factors of MSKAgeMortAccel

We systematically explored the influence of 88 modifiable factors on MSKAgeMortAccel. After Bonferroni correction, 41 factors reached statistical significance (*p* < 5.68 × 10^−4^), with an additional 21 factors showing suggestive associations (5.68 × 10^−4^ < *p* < 0.05) (Table S22).

Linear regression analyses identified air pollution (particulate matter, nitrogen dioxide), negative emotional status (mood swings, miserableness, loneliness, nervousness), adverse early‐life factors (larger childhood body size, maternal smoking), poor physical condition (dental problems), socioeconomic disadvantages (TDI, disability allowance), unhealthy dietary habits (high coffee intake), and harmful behavioral patterns (sleeplessness, smoking, prolonged television watching) as significant predictors of increased MSKAgeMortAccel. Conversely, natural environmental exposure (green spaces), favorable physical conditions (lung function, grip strength), and healthy dietary habits (Healthy Eating Index score, fish and whole grains intake, nutritional supplements) were all associated with decreased MSKAgeMortAccel.

### 2.11 Genetic Determinants for MSKAgeMortAccel

**FIGURE 5:**
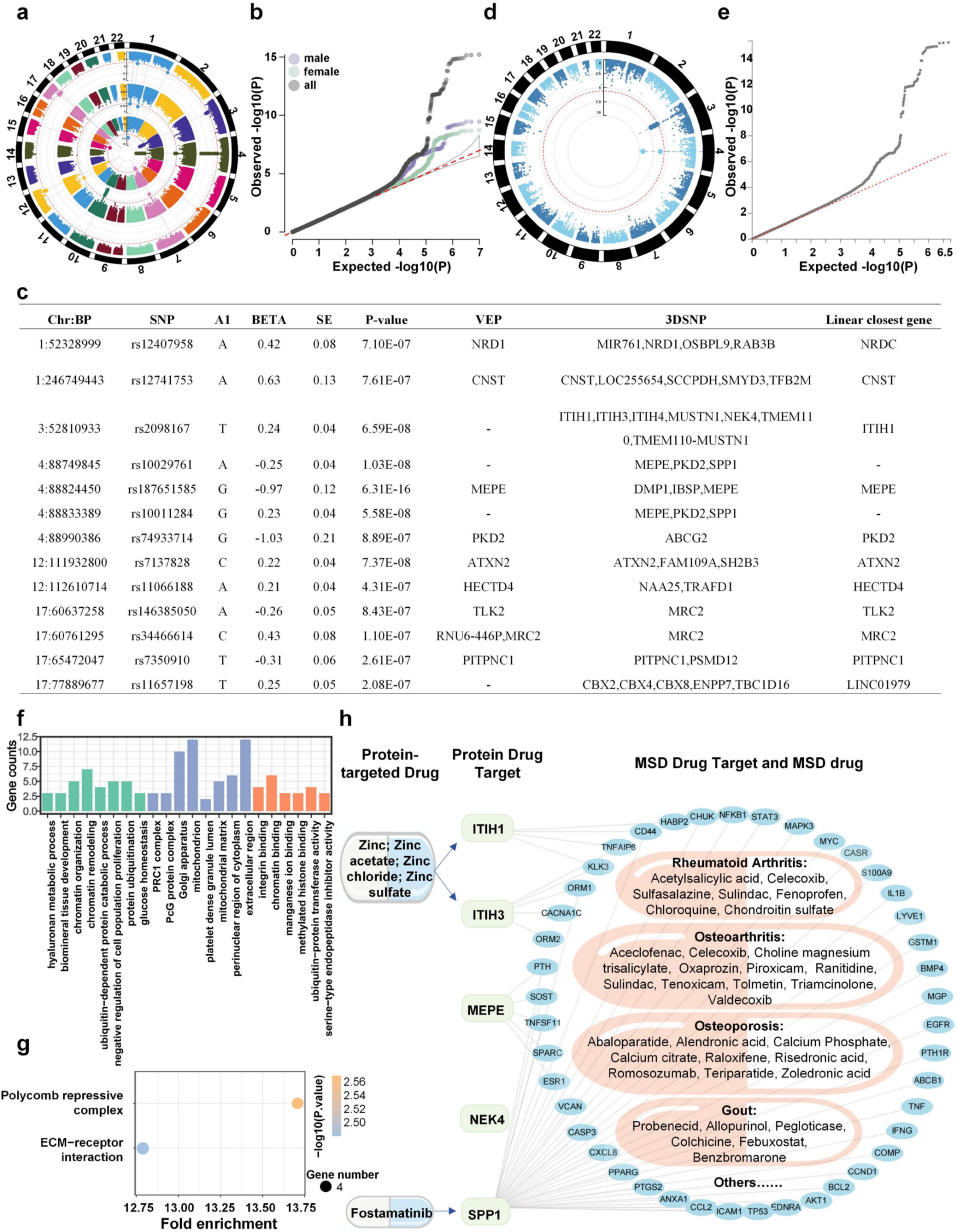
Genetic analysis reveals significant SNPs, candidate genes, pathways, and potential drug targets of MSKAgeMortAccel. (a) Circular Manhattan plot of GWAS for MSKAgeMortAccel. The red dashed line indicates the genome‐wide significance threshold (*p* < 1 × 10^−6^). (b) Q–Q plot of GWAS results. (c) Circular Manhattan plot of GWGAS for MSKAgeMortAccel. The red dashed line indicates the significance threshold (*p* < 0.05/19,146). (d) Q–Q plot of GWGAS results. (e) 13 significant SNPs identified by GWAS (*p* < 1 × 10^−6^). (f) Histogram of GO enrichment analysis. Green represents biological processes, purple represents cell components, and orange represents molecular functions. (g) Bubble chart of KEGG enrichment analysis. (h) Potential drug targets, protein‐targeted drugs, and PPI network. The five proteins coded by key genes concurrently identified through GWAS, FUMA, and MAGMA serve as potential drug targets. Protein‐targeted drugs are identified by Drugbank based on the potential drug targets. MSD drug targets refer to proteins targeted by existing MSD drugs according to Drugbank. The PPI network is presented between potential drug targets and current MSD drug targets. A1, effect allele; BP, base position; Chr, chromosome; GO, Gene Ontology; GWAS, genome‐wide association study; GWGAS, genome‐wide gene association study; KEGG, Kyoto Encyclopedia of Genes and Genomes; MAGMA, Multi‐Marker Analysis of GenoMic Annotation; MSD, musculoskeletal disorder; PPI, protein–protein interaction network; Q–Q plot, quantile–quantile (Q–Q) plot; SNP, single nucleotide polymorphism; VEP, Ensembl Variant Effect Predictor.

We performed a genome‐wide association study (GWAS) on MSKAgeMortAccel in 16,938 European ancestry participants with 9,745,739 variants (Figure S6). A total of 13 independent single nucleotide polymorphisms (SNPs) were significantly associated with MSKAgeMortAccel (*p* < 1 × 10^−6^) (Figure 5a–c, Table S23), three of which reached stronger associations (*p* < 5 × 10^−8^). The strongest SNP was rs187651585 (*p* = 6.31 × 10^−16^), located near *DMP1*, *IBSP*, and *MEPE*, all linked to bone according to their protein function. The second and third most significant SNPs were rs10011284 (*p* = 5.58 × 10^−8^) and rs10029761 (*p* = 1.03 × 10^−8^), both located near *MEPE*, *SPP1*, and *PKD2*, associated with bone formation. Linkage Disequilibrium Score Regression (LDSC) showed no evidence of inflation and systematic bias (intercept = 1.004, λ_GC_ = 1.023), with SNP‐based heritability estimate at 9.42%. To investigate potential sex‐specific effects, we also conducted sex‐stratified GWASs (*N*_Male_ = 7941; *N*_Female_ = 8997), identifying five independent loci in each sex. Genes identified in the overall GWAS, including *MEPE*, *PKD2*, and *SPP1*, showed consistent signals across sexes (Figure 5a–c, Table S23). Concordance analysis of lead SNPs further revealed high agreement in effect estimates (*r* = 0.98–1.00, Figure S7), supporting a shared genetic basis for MSKAgeMortAccel.

Utilizing Functional Mapping and Annotation of GWAS (FUMA v1.5.2) platform, 52 genes potentially related to MSKAgeMortAccel were identified (Table S24). We further conducted a genome‐wide gene association study (GWGAS) using Multi‐Marker Analysis of GenoMic Annotation (MAGMA, v1.08), and 10 genes were identified (*p* < 0.05/19,146) (Figure 5d,e, Table S25). Collectively, GWAS, FUMA, and MAGMA identified 71 candidate genes for downstream analyses.

### 2.12 Protein–Protein Interaction and Functional Enrichment Analyses

Genes that interact physically or functionally may share similar functions in biological processes. The protein–protein interaction (PPI) network for 71 candidate genes of MSKAgeMortAccel was constructed by STRING (v12.0), consisting of 47 nodes and 70 edges. The highest interaction pairs were SPP1‐IBSP, MEPE‐DMP1, IBSP‐DMP1, CBX2‐CBX4, CBX2‐CBX8, and CBX4‐CBX8, each with interaction scores > 0.95. *GNL3*, *ITIH3*, *NEK4*, and *NAA25* were identified as hub genes with connectivity degree > 5 (Figure S8, Tables S26 and S27).

Functional enrichment analyses showed that candidate genes were mainly enriched in pathways related to the epigenetic state of cells (chromatin remodeling/polycomb repressive complex (PRC)) and ECM homeostasis. Classic aging pathways such as ubiquitination, biomineral tissue development, and energy metabolism were also involved (Figure 5f, Table S28). KEGG analyses further supported the results, highlighting PRC and ECM–receptor interaction pathways (Figure 5g, Table S29).

### 2.13 Identification Protein‐Targeted Drugs of MSKAgeMortAccel

GWAS, FUMA, and MAGMA consistently prioritized *ITIH1*, *ITIH3*, *MEPE*, *NEK4*, and *SPP1* as key genetic determinants of MSKAgeMortAccel. Their encoded proteins were considered as potential drug targets. Although none was currently a direct target of approved MSD drugs, ITIH1, ITIH3, MEPE, and SPP1 interacted extensively with 42 proteins targeted by 32 existing MSD drugs (Figure 5h, Tables S30 and S31), suggesting biologically relevant connections to existing MSDs treatment strategies. Further drug repurposing suggested that zinc and its derivatives (targeted ITIH1 and ITIH3) and Fostamatinib (targeted SPP1) hold great promise as candidate drugs for MSDs (Figure 5h).

To further assess the causal relevance of the targets on MSKAgeMortAccel, we performed two‐sample cis‐MR using Inverse Variance Weighted (IVW) and Summary‐data‐based MR (SMR) (Tables S32 and S33). ITIH1, the target of zinc and its derivatives, showed consistent associations with MSKAgeMortAccel in both analyses (β_IVW_ = −0.43, *p*_IVW_ = 1.27 × 10^−5^; β_SMR_ = −0.43, *p*_SMR_ = 4.24 × 10^−7^), with no evidence of heterogeneity (*p*_HEIDI_ = 0.488). ITIH3 also showed significant causal associations (β_IVW_ = 0.24, *p*_IVW_ = 6.35 × 10^−4^; β_SMR_ = 0.33, *p*_SMR_ = 2.04 × 10^−4^), although the HEIDI test indicated possible local genetic heterogeneity or linkage disequilibrium (LD). In contrast, the SPP1 only showed a nominal association in SMR (β_SMR_ = −0.74, *p*_SMR_ = 0.020), while MEPE and NEK4 could not be evaluated due to limited datasets. Together, these findings provide genetic support for selected repurposing candidates, establishing ITIH1 and ITIH3 as causally upstream hubs for targeted intervention.

## 3 Discussion

Using large‐scale proteomics data, combined with organ‐specific enrichment analysis, protein functional annotation, and literature review, we constructed a musculoskeletal protein pool comprising 39 proteins. On this basis, we established two musculoskeletal aging models, MSKAgeMort and MSKAge, to quantify musculoskeletal aging. Musculoskeletal aging acceleration, particularly MSKAgeMortAccel, proved to be a powerful biomarker, significantly predicting all‐cause mortality, MSDs, and other age‐related diseases, while also enhancing the predictive accuracy of conventional mortality risk models. Our analyses further revealed that MSKAgeMortAccel is modulated by modifiable factors, has a heritable basis supported by 13 significant SNPs and 71 candidate genes, is enriched in pathways of epigenetic regulation and ECM interaction, and presents zinc supplementation as a promising repurposable therapeutic. Overall, our study establishes a proteomic framework for assessing musculoskeletal aging and provides a foundation of actionable targets and strategies for healthy aging.

Our study successfully constructed a high‐quality musculoskeletal protein pool through an integrated selection strategy that integrated transcriptomic data with functional annotation and literature review, directly addressing a key limitation of data‐driven methods, which rely on gene expression levels that correlate only modestly (~40%) with protein abundance (de Sousa Abreu et al. 2009; Vogel and Marcotte 2012). Crucially, our selection strategy bridged the gap in bone‐specific proteomic data, which is absent from the GTEx database, thereby ensuring that our clocks capture comprehensive biological signals of both muscle and bone homeostasis. The efficacy of our strategy was supported by the performance of our CA‐based MSKAge, which showed an 87.5% improvement in predictive accuracy (*r* = 0.60) over the existing CA‐based MuscAge (*r* = 0.32) in the overall population (Goeminne et al. 2025). This improvement validates the robustness of our protein selection strategy and establishes a solid foundation for developing a more clinically applicable 2nd‐generation musculoskeletal aging clock.

Our 2nd‐generation musculoskeletal aging model outperformed its 1st‐generation counterpart in predicting mortality, MSDs, and multi‐organ age‐related diseases. The superior performance likely stems from the capacity of mortality data to comprehensively capture systemic physiological decline. While most existing musculoskeletal aging models rely on CA, the prior attempt by Goeminne et al. (2025) to construct a mortality‐based model showed limited predictive power (*r* = 0.26–0.28) and failed to explore its associations with diseases. Our 2nd‐generation musculoskeletal aging model makes up for these defects by achieving a higher correlation (*r* = 0.92 in the overall population) and generating an acceleration metric that robustly predicts mortality and MSDs. Moreover, comparisons with published proteomic musculoskeletal aging clocks showed that MSKAgeMort and MSKAgeMortAccel provided stronger risk associations and better predictive performance, while improving conventional risk models. These advancements position MSKAgeMort as a reliable tool for quantifying musculoskeletal aging.

From a pathophysiological perspective, organ‐specific aging affects target organ function, leading to chronic disease onset (Tian et al. 2023). However, organ aging extends beyond a localized process, exerting cascading effects through multi‐organ interactions and manifesting as systemic physiological decline. As expected, our findings confirm an important role of the musculoskeletal system in maintaining bodily homeostasis, as its senescence is strongly correlated with both a spectrum of geriatric diseases and systemic aging. Therefore, by providing precise quantification of organ aging while maintaining the capacity to capture systemic aging, the integration of our musculoskeletal aging models with conventional approaches enables a comprehensive assessment of aging.

Our findings identify multiple factors that influence musculoskeletal aging. Specifically, harmful exposures including air pollutants, negative psychological status, and adverse early‐life experiences, may accelerate musculoskeletal aging through oxidative stress, NF‐κB‐mediated inflammation (Zhang et al. 2024), glucocorticoid‐driven bone loss (Cizza et al. 2010, 2001; Foertsch et al. 2017), and epigenetic alterations (Gim et al. 2023; Goodrich et al. 2016; Visconti et al. 2021). Conversely, protective factors, including green spaces exposure and healthy diets, may decelerate musculoskeletal aging (Dominguez et al. 2025; Veronese et al. 2019, 2017; Welch et al. 2025) by mitigating environmental damage (de Vries et al. 2025), modulating immune responses (Bailey and Holscher 2018), promoting anti‐inflammatory metabolites (i.e., short‐chain fatty acids) (Pagliai et al. 2020), and maintaining a favorable gut microbiota composition (Merra et al. 2020). These findings support the concept that biological aging is regulated through an integrated network involving inflammation, oxidative stress, endocrine signaling, epigenetic remodeling, and metabolic homeostasis. The rate of musculoskeletal aging reflects a dynamic balance between risk and resilience factors, underscoring the value of targeted lifestyle and environmental interventions in promoting healthy aging. Notably, alcohol consumption frequency showed a protective effect on MSKAgeMortAccel. This observation may reflect the demographic characteristics of the UKB, which predominantly consists of relatively healthy and wealthy individuals, where alcohol consumption may serve as a proxy for social engagement and socioeconomic status. Moreover, the alcohol consumption in UKB did not reach an abuse level, and daily alcohol consumption conferred no discernible benefits to MSKAgeMortAccel.

Genetic analyses identified key genetic determinants of MSKAgeMortAccel. *MEPE*, *SPP1*, and *PKD2* showed consistent signals in overall and sex‐stratified GWAS. These genes are closely related to bone homeostasis, as *MEPE* and *SPP1* regulate bone ECM, while *PKD2* modulates osteoblastogenesis, thereby influencing bone turnover (Xiao et al. 2014), suggesting that bone metabolism represents a shared genetic feature of MSKAgeMortAccel across sexes. Interestingly, sex‐specific patterns were also observed, with female‐specific signals enriched in bone mineralization and male‐specific loci implicated in inflammatory immune responses. Additionally, the larger effect magnitude observed in males (slope > 1, *p* < 0.05) suggests that sex‐specific physiological environments may modulate the genetic effects. Beyond genetic loci, FUMA and MAGMA analyses additionally highlighted the key roles of *ITIH1*, *ITIH3*, and *NEK4* in accelerating musculoskeletal aging. *ITIH1* and *ITIH3* appear to modulate musculoskeletal homeostasis through ECM remodeling (Austin et al. 2024; Zou and Shao 2024), whereas *NEK4* regulates replicative senescence and DNA damage response (Nguyen et al. 2012). Furthermore, PPI also highlighted *NAA25* and *GNL3* as hub genes involved in cell‐cycle regulation, apoptosis, and DNA damage response (Lebdy et al. 2023; Xu et al. 2021), with genetic variants in these genes previously linked to RA and OA (B. Liu et al. 2018; Prahalad et al. 2009; Wang et al. 2022).

Functional enrichment analyses at both protein and gene levels emphasized ECM homeostasis, cytoskeletal organization, and epigenetic regulation as central mechanisms of musculoskeletal aging. At the protein level, shared CA‐ and mortality‐associated signatures converged on muscle structure and contraction, skeletal development, ECM homeostasis, and cytoskeleton‐related pathways, supporting a conserved biological foundation. Consistently, gene‐level enrichment further identified ECM–receptor interaction, reinforcing the central role of ECM remodeling and cell–matrix communication. In addition, epigenetic regulation, particularly PRC, was also highlighted. Dysregulation of these pathways may impair cell adhesion, migration, differentiation, and survival, ultimately compromising the mechanical properties and regenerative capacity of bone and muscle. In addition, ubiquitination also emerged as a key process, as it regulates muscle protein degradation, mitochondrial quality, and neuromuscular signaling (Hughes et al. 2022). Its dysfunction may lead to the collapse of protein homeostasis, directly driving sarcopenia and bone metabolic abnormalities.

From a therapeutic perspective, the five proteins (ITIH1, ITIH3, MEPE, NEK4, and SPP1) encoded by key genes identified by our integrated approaches serve as potential drug targets. Although no approved MSD drugs target these proteins, their extensive interactions with established MSD drug targets underscore therapeutic potentials. Drug repurposing identified Fostamatinib and zinc as candidate agents for MSDs treatment. Notably, the zinc‐related targets, ITIH1 and ITIH3, were supported by two‐sample cis‐MR and SMR, providing genetic evidence for their involvement in musculoskeletal aging. Compared with fostamatinib, a thrombocytopenia drug requiring prescription and remaining an aggressive therapeutic strategy, zinc and its derivatives are widely accessible as nutritional supplements. Mechanistically, zinc promotes collagen matrix synthesis, bone mineralization, and bone turnover (Amin et al. 2020; Molenda and Kolmas 2023) by promoting proliferation and differentiation of osteoblasts and inhibiting osteoclast generation and inducing their apoptosis (Seyedmajidi et al. 2014). Furthermore, zinc deficiency disrupts the protein homeostasis and mitochondrial function of skeletal muscle, affecting muscle mass and strength (Reddy et al. 2022). These mechanisms resonate to highlight the broad prospects of zinc in managing MSDs. Collectively, our results bridge genetic insights with therapeutic potentials, providing a basis for future studies targeting musculoskeletal aging.

This study has several limitations. First, the UKB proteomics data did not cover all musculoskeletal proteins. Although our aging clocks demonstrated satisfying predictive performance, it is recommended that the models be thoroughly validated when applied across datasets. Second, the cross‐sectional design limited causal inferences on the roles of modifiable factors in musculoskeletal aging. Third, although baseline disease exclusion reduced confounding by overt disease, it may introduce selection bias, limiting generalizability to populations with higher disease burden and reducing statistical power in downstream analyses, including GWAS. Fourth, UKB is not fully representative of either the global population or the UK population, as participants are predominantly healthy, socioeconomically advantaged, and white British. Consequently, the generalizability of our findings to other populations should be interpreted with caution, and validation in diverse multi‐ethnic cohorts is warranted. Finally, future studies should incorporate more musculoskeletal‐specific phenotypes or biomarkers to optimize model construction, and experimental work is needed to validate the identified molecular mechanisms, drug targets, and repurposed drugs.

## 4 Conclusion

We have successfully developed and validated two proteomic‐based models, MSKAgeMort and MSKAge, which provide robust quantitative measures of musculoskeletal aging. Both models significantly outperform conventional risk factors and existing clocks in predicting all‐cause mortality, MSDs, and age‐related diseases. Air pollution, negative emotions, poor health conditions, and unhealthy lifestyles significantly accelerate musculoskeletal aging. Mechanistically, musculoskeletal aging acceleration is driven by the synergistic dysregulation of epigenetic and impaired ECM–receptor. Zinc and its derivatives may act as promising therapeutic agents. Together, this work establishes a comprehensive framework for assessing musculoskeletal aging and provides a foundation for early risk detection and targeted interventions to preserve mobility and independence in an aging population.

## 5 Online Methods

### 5.1 Study Participants

UKB is a large prospective cohort enrolling > 500,000 participants aged 37–73 years, recruited from 22 assessment centers in England, Scotland, and Wales between 2006 and 2010. Data collection encompassed detailed personal information, anthropometric data, medical records, and biological samples.

### 5.2 Proteomics Data

Baseline blood samples collected at recruitment were profiled using the Olink Explore 3072 proximity extension assay in ~54,000 UKB participants, which successfully measured 2923 proteins. After quality control and batch effect correction, normalized protein expression (NPX) values were generated for each participant. NPX is Olink's relative protein quantification unit on a log2 scale.

### 5.3 Inclusion and Exclusion Criteria

Participants with > 30% missing values and proteins with > 30% missing values were removed from the analysis, retaining 44,782 participants and 2920 proteins. The proteomics dataset was then imputed using the k‐nearest neighbors algorithm (*k* = 10). Based on 44,782 participants, a further screening process was conducted. The exclusion criteria were as follows: (1) missing important variables (CA, sex, and death information); (2) patients with history of MSDs or diseases affecting the musculoskeletal system, including skeletal development disorders, disorders of muscles, fracture, hyperparathyroidism, hypothyroidism, hyperthyroidism, Cushing's syndrome, CKD, cancer, diabetes, hyperlipidemia, vitamin D deficiency, malnutrition, anorexia, heart failure, hypertension, CVD, cerebrovascular disease, etc. This screening process, detailed in Figure S1, included 21,070 participants to reduce confounding and ensure data completeness.

### 5.4 Musculoskeletal Protein Pool Construction

Our musculoskeletal protein pool contained muscle‐enriched proteins and bone‐enriched proteins. All proteins were selected from the UKB proteomics, and the screening criteria were based on organ enrichment in gene expression databases, functional annotation, and literature expertise.

For muscle‐enriched proteins, transcriptomic data was used to identify candidates as transcriptomics informs the organ of origin of a protein. Specifically, based on GTEx, a protein was considered muscle‐enriched if its average GTEx gene expression in muscle was at least four times higher than in all other organs. To enrich the protein pool, we used UniProt (https://www.uniprot.org/) to functionally annotate proteins available in the UKB proteomics regardless of their tissue‐specific GTEx gene expression levels. Meanwhile, literature search was also conducted to understand the functional roles of these proteins. A protein was considered a muscle‐enriched protein, thus added to our pool only if it exerted biological functions related to skeletal muscle confirmed by both UniProt and literature search.

Given an absence of bone tissue data in GTEx, bone‐enriched proteins were identified by integrating UniProt annotation and literature evidence.

### 5.5 Development of Musculoskeletal Aging Clocks

Participants were randomly split into training (70%) and testing (30%) sets for each sex. All models were trained separately for females and males (Figure S1). Analyses were performed using R packages survival (v3.8.3), glmnet (v4.1.8), flexsurv (2.3.2), and caret (v7.0.1).

Development of MSKAgeMort: MSKAgeMort was trained in two steps. First, mortality‐associated proteins were identified. Initially, each protein was tested for association with all‐cause mortality per 1‐SD increment using sex‐stratified Cox regression. An FDR threshold of < 0.2 yielded 29 candidate proteins (21 female‐specific, 25 male‐specific, 17 overlapping). These proteins, together with CA, were then included in sex‐stratified Lasso‐Cox models in the training set. Through 10‐fold cross‐validation, the selection was optimized, yielding 12 variables for females and 17 for males, both including CA. Second, following Levine et al. (2018), two proportional hazards regression models based on the parametric Gompertz distribution were fitted: a multivariate model with selected variables and a CA‐only model. The 10‐year all‐cause mortality risk was estimated from both models. By equating their risks and solving for age, mortality risk was converted to years, giving rise to MSKAgeMort. The established formulas were then applied in the testing set.

Development of MSKAge: MSKAge was trained in two steps. First, CA‐associated proteins were identified. The effects of the plasma proteins (per 1‐SD increment) on CA were assessed by sex‐stratified linear regression. An FDR threshold of < 0.2 yielded 32 candidate proteins (30 female‐specific, 30 male‐specific, 28 overlapping). Second, LASSO with 10‐fold cross‐validation was used to construct MSKAge within the training set. The established formulas were then used in the testing set.

Model performance was evaluated by *r* and MAE. MSKAgeMortAccel and MSKAgeAccel were defined as residuals from linear regressing each on CA, following Liu (2021).

### 5.6 Covariates

All analyses were adjusted for sex, and sex‐stratified analyses were additionally performed where appropriate. Covariates included CA (year), sex (female or male), ethnicity (White or Non‐white), TDI, BMI (kg/m^2^), education level (higher education level, less than college or university degree), alcohol consumption (never, previous, or current), smoking status (never, previous, or current), physical activity (low, moderate, high), and sleep duration (< 7 h, 7–8 h, > 8 h). Missing values, including do not know and prefer not to answer, were set to NA and imputed using Multiple Imputation by Chained Equations (mice v3.17.0 R package) with the Random Forest function.

### 5.7 Health‐Related Outcomes

Mortality data came from national death registries, with time‐to‐death defined by UKB field 40,000. Follow‐up person‐years were defined from the date of baseline to the date of death, loss of follow‐up, or end of follow‐up (England: December 31, 2022; Wales: May 31, 2022; Scotland: August 31, 2022), whichever came first.

Disease diagnoses were identified by ICD‐9/10 codes (Table S34). We identified MSDs, with a focus on five common conditions, RA, OA, LBP, NP, and gout. Additionally, age‐related diseases were categorized across major systems, endocrine and metabolic diseases (e.g., type 2 diabetes, hyperlipidemia), circulatory system (e.g., CVD, hypertension), digestive system (e.g., liver diseases, cirrhosis), respiratory system (e.g., COPD), renal system (e.g., CKD), and nervous system (e.g., AD, depression). Follow‐up person‐years were defined from the date of baseline to the incident diseases, date of death, loss of follow‐up, or end of follow‐up, whichever came first.

### 5.8 Environmental Factors

We explored 88 potentially modifiable factors, classifying (1) natural environmental factors (e.g., PM2.5, greenspace percentage); (2) psychosocial factors (e.g., mood swings, miserableness); (3) socioeconomic factors (e.g., TDI); (4) medical history and early life factors (e.g., comparative body size at age 10); (5) physiological indicators (e.g., grip strength); (6) lifestyle factors (e.g., diet, smoking) on MSKAgeMortAccel. Details please refer to Table S22 and elsewhere (Jia et al. 2024).

### 5.9 Study Approval

All participants provided written informed consent, approved by the North West Multicenter Research Ethics Committee. The deidentified data required no additional ethical approval.

### 5.10 Statistical Analysis

#### 5.10.1 Basic Characteristics of Participants

Baseline characteristics were described using mean ± SD or median (IQR) for continuous variables, and count (percentage) for categorical variables. Group differences were examined using the Kruskal–Wallis test for continuous variables, and chi‐squared (*χ* ^2^) or Fisher's exact test for categorical variables.

#### 5.10.2 Associations of MSKAgeAccel/MSKAgeMortAccel With Physical Health Conditions

Multivariable Cox proportional hazards models were used to estimate associations between MSKAgeAccel/MSKAgeMortAccel and incident outcomes. Proportional hazards assumptions were tested by scaled Schoenfeld residuals. Prevalent cases were excluded for each outcome. The main model adjusted for CA, sex, ethnicity, TDI, education level, physical activity, BMI, smoking status, alcohol status, and sleep duration. KM survival curves were also generated for all‐cause mortality and MSDs across MSKAgeAccel/MSKAgeMortAccel quartiles and compared by log‐rank test. Several sensitivity analyses were conducted across sexes and training–testing sets. All *p* values were two‐sided, with significance set at *p* < 0.05. ROC curves were generated using timeROC (v0.4) to compare CA‐only, MSKAge‐only/MSKAgeMort‐only, conventional risk factors (CA, sex, ethnicity, TDI, education level, physical activity, BMI, smoking, and alcohol consumption status), and combined (conventional + MSKAge/MSKAgeMort) models for 10‐year all‐cause mortality prediction.

#### 5.10.3 Sensitivity Analysis

We performed a sensitivity analysis by excluding individuals who died or developed diseases affecting the musculoskeletal system within 2 years after baseline. The entire analytical pipeline, including proteomic feature selection, model construction, and downstream association analyses, was repeated in this restricted cohort.

#### 5.10.4 Comparisons With Existing Proteomic Musculoskeletal Aging Clocks

We compared the predictive performance of MSKAgeMort with three published proteomic musculoskeletal aging clocks: (1) a CA‐based LASSO model developed by Oh et al. (2025), and (2) two elasticnet models trained on CA and mortality developed by Goeminne et al. (2025). The published clocks were reconstructed using reported protein weights. Biological age acceleration was calculated for each clock as residual from regressing predicted biological age on CA. We then compared MSKAgeMort with reconstructed published clocks, and compared MSKAgeMortAccel with corresponding acceleration measures. Spearman correlations assessed agreement across clocks. Predictive performance for all‐cause mortality and MSDs was evaluated by C‐index. Cox regression models using standardized biological age and acceleration metrics were further applied to compare risk associations across models.

#### 5.10.5 Environmental Factors Influencing MSKAgeMortAccel

Multivariable linear regression models were used to identify modifiable factors that correlate with MSKAgeMortAccel. Bonferroni significance was set at *p* < 0.05/88 = 5.68 × 10^−4^, while 5.68 × 10^−4^ < *p* < 0.05 were considered suggestive. Model 1 adjusted for CA, sex, and ethnicity, and model 2 additionally adjusted for BMI.

#### 5.10.6 GWAS

GWASs were performed using imputed genotype data from UKB. Participants with low genetic call rates, high heterozygosity, genetic sex inconsistent with self‐reported sex, sex chromosome aneuploidy, or excess relatives were excluded. Analyses were restricted to European ancestry individuals. Variant‐level quality control retained (1) SNPs in autosome, (2) minor allele frequency ≥ 0.01, and (3) Hardy–Weinberg equilibrium test *p* ≥ 1 × 10^−6^. A total of 16,938 participants and 9,745,739 variants passed quality control (Figure S4). Sex‐stratified GWASs were further conducted in males (*N* = 7941) and females (*N* = 8997). GWASs were performed using PLINK (v2.0), adjusting for sex, CA, genotyping array, and the top 10 principal components. Independent lead variants were defined as genome‐wide significant variants (*p* < 1 × 10^−6^) that were not in LD with a more significant variant at *r* ^2^ ≥ 0.1 within a 500 kb window. LDSC (v1.0.1) was used to assess genomic inflation and SNP‐based heritability. Functional annotation was performed using VEP (https://grch37.ensembl.org/info/docs/tools/vep/) and 3DSNP (https://omic.tech/3dsnpv2/). VEP assigns genes by physical proximity, whereas 3DSNP annotates SNP function based on 3D SNP‐gene interactions.

#### 5.10.7 Functional Mapping and Annotation and MAGMA

FUMA (https://fuma.ctglab.nl/home) was used for functional mapping and annotation of the overall GWAS, with the European 1000 Genomes Phase 3 panel as population reference and GRCh37/hg19 as the genome build. FUMA takes GWAS association summary results as input, integrates multiple‐source information and facilitates post‐GWAS analysis, such as functional annotation and gene prioritization. The genome‐wide significance threshold was set at *p* < 1 × 10^−6^, and other parameters were kept as default. Subsequently, positional mapping was conducted to connect risk loci to nearby genes within 10 kb. Moreover, GWGAS analysis was performed using MAGMA v1.08 across 19,146 protein‐coding genes, with Bonferroni significance set at *p* < 0.05/19,146. Genes identified by any of GWAS, FUMA, or MAGMA (*N* = 71) were designated as candidate genes for PPI or pathway analysis, while genes confirmed by all three approaches (*N* = 5) were designated as candidate genes for drug target analysis.

#### 5.10.8 Protein–Protein Interaction Analysis

STRING (v12.0, https://cn.string‐db.org) was used to construct a PPI network for 71 candidate genes. Based on the interaction scores generated from multi‐source data (experimentally determined interactions, database‐annotated information, and automated text mining knowledge), we screened out PPI pairs with interaction scores > 0.4. Subsequently, the Cytoscape software was used to generate a visual network of PPI pairs, and the CytoHubba plugin was employed to calculate node connectivity. Eventually, we considered 4 genes as hub genes crucial in the PPI network with connectivity degree > 5.

#### 5.10.9 GO and KEGG Enrichment Analysis

DAVID Bioinformatics Resources (https://davidbioinformatics.nih.gov/) was used to conduct GO and KEGG enrichment analyses on CA−/mortality‐associated proteins and 71 candidate genes to obtain detailed information on cell components, molecular functions, biological processes, and KEGG pathways. *p* < 0.05 was considered significant.

#### 5.10.10 Identification Protein‐Targeted Drugs of MSKAgeMortAccel

Potential drug targets (*N* = 5) were defined as those encoded by genes that successfully passed all three analyses (GWAS, FUMA, and MAGMA). We first searched the DrugBank (https://go.drugbank.com/) database to identify (1) existing MSD drugs and their protein targets, and (2) existing other drugs. We then performed “look‐up” or PPI analysis to confirm if the identified potential druggable proteins were targets of (1) existing MSD drugs, or (2) existing other drugs, or interacted with known protein targets of existing MSD drugs. MSD drugs included approved therapeutics for RA, OA, gout, OP, and other MSDs. STRING was used to assess interactions between potential drug targets and known MSD drug targets. Only interactions with a confidence score greater than 0.4 were retained to ensure biologically meaningful associations.

#### 5.10.11 Two‐Sample Cis‐MR and SMR

Two‐sample cis‐MR was performed using TwoSampleMR (v0.7.5). Independent cis‐pQTLs located within ±1 Mb of the transcription start site were selected as instrumental variables (IVs) using *p* < 5 × 10^−8^ and clumped with the following parameters: —clump‐p1 5e‐8 —clump‐p2 1e‐5 —clump‐r2 0.01 —clump‐kb 1000. For proteins with multiple IVs, IVW was used; otherwise, the Wald ratio was applied. SMR (v1.3.1), with the HEIDI test was further used to assess whether observed associations were consistent with shared causal/pleiotropic signals rather than LD. Bonferroni‐corrected *p* < 0.05/3 was considered significant.

## Author Contributions

**Yangdan Zhong:** formal analysis, methodology, software, conceptualization, investigation, visualization, writing – original draft preparation, data curation. **Maoyao Xia:** formal analysis, methodology, software, conceptualization, investigation, visualization, writing – review and editing, data curation. **Xunying Zhao:** formal analysis, investigation, visualization, writing – review and editing. **Yang Qu:** investigation, visualization, writing – review and editing. **Bowen Lei:** investigation, visualization, writing – review and editing. **Sirui Zhen:** investigation, visualization, writing – review and editing. **Tao Han:** investigation, visualization, writing – review and editing. **Rong Xiang:** investigation, visualization, writing – review and editing. **Jinyu Xiao:** investigation, visualization, writing – review and editing. **Xin Song:** investigation, visualization, writing – review and editing. **Xiaofeng Ma:** investigation, visualization, writing – review and editing. **Bin Yang:** investigation, visualization, writing – review and editing. **Di Zhang:** investigation, visualization, writing – review and editing. **Jinyu Zhou:** investigation, visualization, writing – review and editing. **Zilan Chen:** investigation, visualization, writing – review and editing. **Yuqi Pang:** investigation, visualization, writing – review and editing. **Ye Ju:** investigation, visualization, writing – review and editing. **Ting Liu:** investigation, visualization, writing – review and editing. **Zihao Li:** investigation, visualization, writing – review and editing. **Lu Long:** writing – review and editing. **Tao Zhang:** writing – review and editing. **Jiayuan Li:** data curation, writing – review and editing. **Mengyu Fan:** writing – review and editing. **Zuyun Liu:** data curation, writing – review and editing. **Xia Jiang:** conceptualization, methodology, investigation, visualization, project administration, supervision, writing – review and editing, funding acquisition.

## Funding

This study was supported by the Recruitment Program for Young Professionals of China, the Science Fund for Creative Research Groups of Science and Technology Bureau of Sichuan Province (2024NSFTD0030), the Natural Science Foundation of Sichuan Province, China (2025ZNSFSC1775), Sichuan Medical Association (S21003), National Natural Science Foundation of China (82171584, 72374180), “Pioneer” and “Leading Goose” R&D Programs of Zhejiang Province (2025C02104, 2023C03163), Zhejiang Key Laboratory of Intelligent Preventive Medicine (2020E10004), and Zhejiang University School of Public Health Interdisciplinary Research Innovation Team Development Project. The sponsors of this study had no role in study design, data collection, analysis, interpretation, writing of the report, or the decision for submission.

## Conflicts of Interest

The authors declare no conflicts of interest.

## Supporting information


**Figure S1:** The flowchart of participants selection in this study. Diseases we excluded include: Musculoskeletal diseases, skeletal development disorders, disorders of muscles, fracture, hyperparathyroidism, hypothyroidism, hyperthyroidism, Cushing's syndrome, chronic kidney disease, cancer, diabetes, hyperlipidemia, vitamin D deficiency, malnutrition, anorexia, heart failure, hypertension, cardiovascular disease, cerebrovascular disease, etc.
**Figure S2:** Venn plot showing the number of musculoskeletal proteins associated with (a) mortality and (b) CA in different sexes. Red represents female and green represents male.
**Figure S3:** Distributions of CA, MSKAge and MSKAgeMort, MSKAgeAccel and MSKAgeMortAccel, and their correlations. CA, chronological age; MSKAgeAccel, MSKAge acceleration; MSKAgeMortAccel, MSKAgeMort acceleration.
**Figure S4:** Enrichment analyses of shared and sex‐specific musculoskeletal protein signatures associated with CA and mortality. (a) GO enrichment analysis. (b) KEGG pathway enrichment analysis. (A) Proteins shared between males and females associated with CA; (B) Proteins shared between males and females associated with mortality; (C) Female‐specific proteins associated with mortality; (D) Male‐specific proteins associated with mortality. In a, GO terms are grouped into biological process (BP), cellular component (CC), and molecular function (MF).
**Figure S5:** Sensitivity analysis of musculoskeletal aging models after excluding participants with early incident diseases or death within two years after baseline. (a, b) Associations between proteins and all‐cause mortality in the sensitivity analysis for females and males, respectively. (c, d) Concordance of hazard ratios for mortality‐associated proteins between the original and sensitivity analyses. (e, f) Associations between proteins and chronological age in the sensitivity analysis for females and males, respectively. (g, h) Concordance of regression coefficients for chronological age‐associated proteins between the original and sensitivity analyses. (i, j) Correlation of model coefficients for the 2nd‐generation musculoskeletal aging model, MSKAgeMort, between the original and sensitivity analyses in females and males. (k, l) Correlation of model coefficients for the 1st‐generation musculoskeletal aging model, MSKAge, between the original and sensitivity analyses in females and males. (c–l) With inset Venn diagrams showing the overlap of selected proteins. (m, n) Correlations between predicted MSKAgeMort and chronological age in females and males. (o, p) Correlations between predicted MSKAge and chronological age in females and males. (q, r) Kaplan–Meier survival curves according to aging acceleration groups derived from MSKAgeMort and MSKAge, respectively. (s) Receiver operating characteristic curves comparing the predictive performance of chronological age, MSKAge, MSKAgeMort, and combined models for all‐cause mortality. (t) Associations of MSKAgeAccel and MSKAgeMortAccel with all‐cause mortality and musculoskeletal outcomes in the sensitivity cohort. Error bars represent 95% confidence intervals. CA, chronological age; HR, hazard ratio; MAE, mean absolute error; MSD, musculoskeletal disorders; NP, neck pain; OA, osteoarthritis; RA, rheumatoid arthritis.
**Figure S6:** Flowchart of GWAS quality control processes. GWAS, genome‐wide association study; HWE, Hardy–Weinberg equilibrium; MAF, Minor Allele Frequency; UKB, UK Biobank.
**Figure S7:** Concordance of genetic effect estimates across overall and sex‐stratified GWAS of MSKAgeMortAccel. (a) Correlation of SNP effect estimates between the overall GWAS and female‐stratified GWAS. (b) Correlation of SNP effect estimates between the overall GWAS and male‐stratified GWAS. (c) Correlation of SNP effect estimates between female‐ and male‐stratified GWAS. Points represent independent lead SNPs, the solid line represents the fitted regression line, the shaded area indicates the 95% confidence interval, and the dashed line indicates the line of equality. Pearson correlation coefficients and corresponding *p* values are shown in each panel.
**Figure S8:** Protein–protein interaction network of candidate genes of MSKAgeMortAccel. The circle with deeper color indicating a higher connectivity degree of gene. Dark blue represents the hub genes. MAGMA, Multi‐Marker Analysis of GenoMic Annotation.

**Data S1:** acel70636‐sup‐0002‐supinfo.xlsx.

## Data Availability

Data sharing does not apply to this article as no new data were created or analyzed in this study. Full information on how to access UK Biobank data can be found on its website (https://www.ukbiobank.ac.uk/). More details of the approaches as well as the codes are available at https://www.finngen.fi/en (FinnGen), https://opengwas.io/ (OpenGWAS), https://www.uniprot.org/ (UniProt), https://github.com/cran/glmnet (glmnet), https://github.com/amices/mice (mice), https://www.cog‐genomics.org/plink/2.0 (plink 2.0), https://grch37.ensembl.org/info/docs/tools/vep/ (VEP), https://omic.tech/3dsnpv2/ (3DSNP), https://fuma.ctglab.nl/home (FUMA), https://cn.string‐db.org (STRING), https://cytoscape.org (Cytoscape), https://davidbioinformatics.nih.gov/ (DAVID), https://go.drugbank.com/ (Drugbank). Details are provided in Supporting Information Methods. The core analysis scripts used for constructing MSKAge and MSKAgeMort and for conducting downstream statistical analyses have been curated and organized. Code is available upon reasonable request from the corresponding author.
