# Supplementary material for: Construction and Validation of Plasma Protein‐Based Musculoskeletal Biological Age and Genetic and Environmental Risk Profiles
Source: Aging Cell. 2026 Jul 25;25(8):e70636. doi: 10.1111/acel.70636 (PMC13401616; doi:10.1111/acel.70636)
Supplement: Supplementary file 1 — Figure S1: The flowchart of participants selection in this study. Diseases we excluded include: Musculoskeletal diseases, skeletal development disorders, disorders of muscles, fracture, hyperparathyroidism, hypothyroidism, hyperthyroidism, Cushing's syndrome, chronic kidney disease, cancer, diabetes, hyperlipidemia, vitamin D deficiency, malnutrition, anorexia, heart failure, hypertension, cardiovascular disease, cerebrovascular disease, etc. Figure S2: Venn plot showing the number of musculoskeletal proteins associated with (a) mortality and (b) CA in different sexes. Red represents female and green represents male. Figure S3: Distributions of CA, MSKAge and MSKAgeMort, MSKAgeAccel and MSKAgeMortAccel, and their correlations. CA, chronological age; MSKAgeAccel, MSKAge acceleration; MSKAgeMortAccel, MSKAgeMort acceleration. Figure S4: Enrichment analyses of shared and sex‐specific musculoskeletal protein signatures associated with CA and mortality. (a) GO enrichment analysis. (b) KEGG pathway enrichment analysis. (A) Proteins shared between males and females associated with CA; (B) Proteins shared between males and females associated with mortality; (C) Female‐specific proteins associated with mortality; (D) Male‐specific proteins associated with mortality. In a, GO terms are grouped into biological process (BP), cellular component (CC), and molecular function (MF). Figure S5: Sensitivity analysis of musculoskeletal aging models after excluding participants with early incident diseases or death within two years after baseline. (a, b) Associations between proteins and all‐cause mortality in the sensitivity analysis for females and males, respectively. (c, d) Concordance of hazard ratios for mortality‐associated proteins between the original and sensitivity analyses. (e, f) Associations between proteins and chronological age in the sensitivity analysis for females and males, respectively. (g, h) Concordance of regression coefficients for chronological age‐associated p [file ACEL-25-e70636-s001.docx]

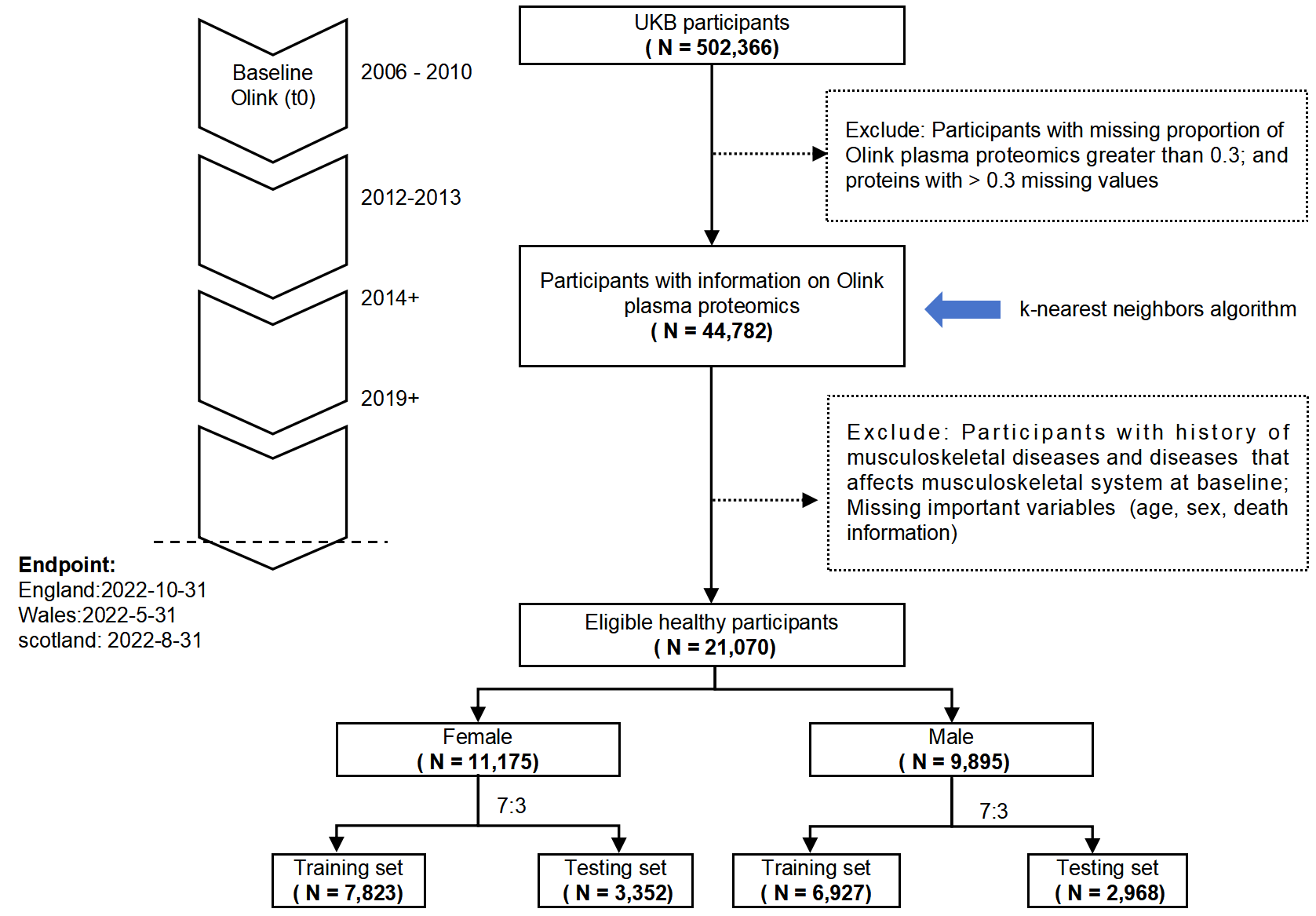
**Supplementary Figure 1**. **The flowchart of participants selection in this study**. Diseases we excluded include: Musculoskeletal diseases, Skeletal Development Disorders, Disorders of muscles, Fracture, Hyperparathyroidism, Hypothyroidism, Hyperthyroidism, Cushing's syndrome, Chronic Kidney Disease, Cancer, Diabetes, Hyperlipidemia, Vitamin D deficiency, Malnutrition, Anorexia, Heart failure, Hypertension, Cardiovascular disease, Cerebrovascular disease, etc.


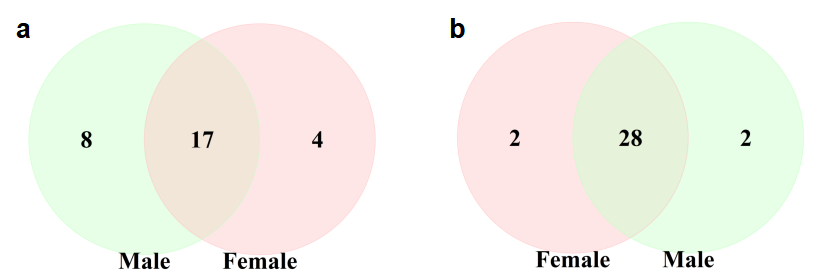


**Supplementary Figure 2. Venn plot showing the number of musculoskeletal proteins associated with a mortality and b CA in different sexes.** Red represents female and green represents male.


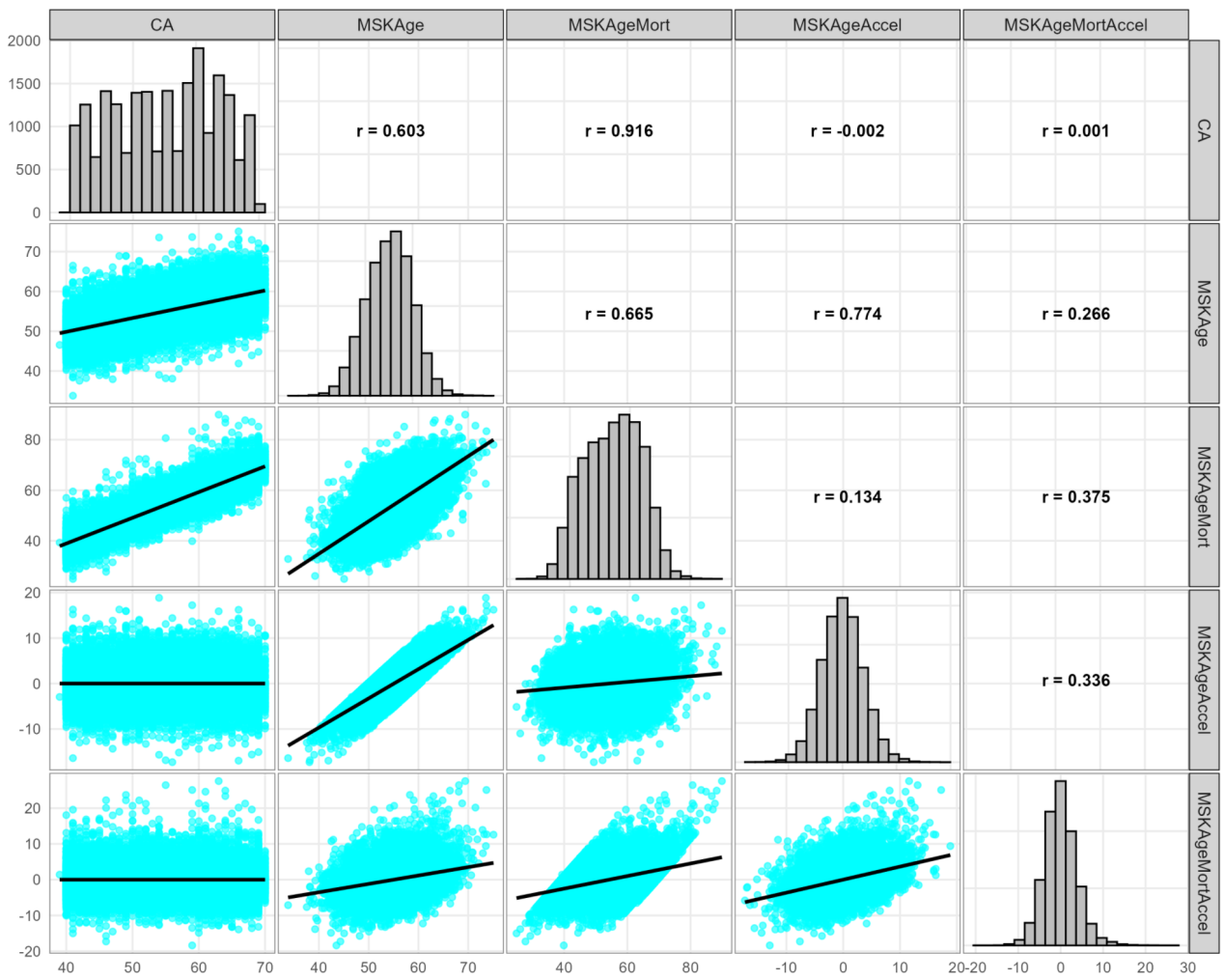


**Supplementary Figure 3. Distributions of CA, MSKAge and MSKAgeMort, MSKAgeAccel and MSKAgeMortAccel, and their correlations.** CA, Chronological age; MSKAgeMortAccel, MSKAgeMort acceleration; MSKAgeAccel, MSKAge acceleration.


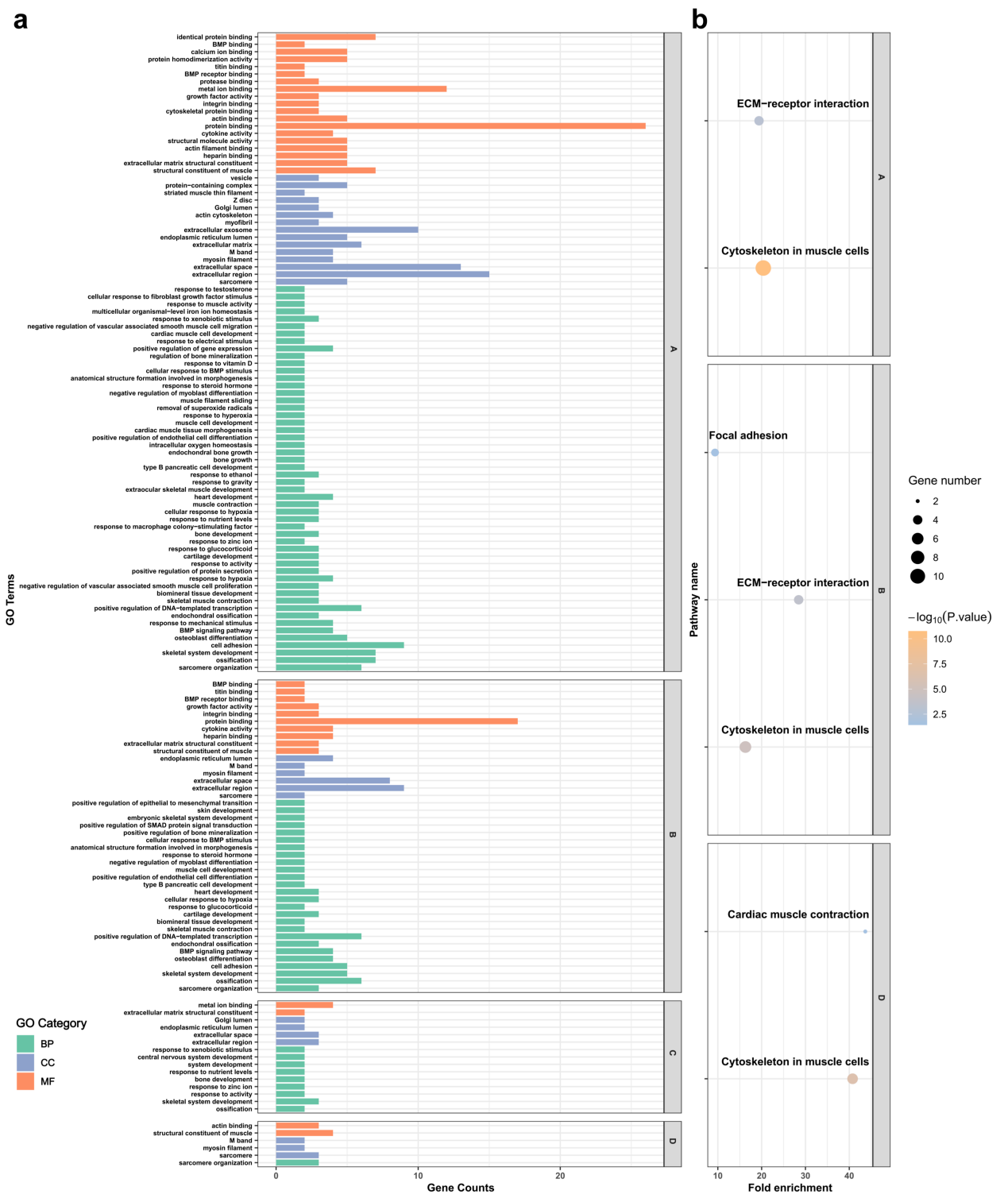


**Supplementary Figure 4. Enrichment analyses of shared and sex-specific musculoskeletal protein signatures associated with CA and mortality. a** GO enrichment analysis. **b** KEGG pathway enrichment analysis. A, proteins shared between males and females associated with CA; B, proteins shared between males and females associated with mortality; C, female-specific proteins associated with mortality; D, male-specific proteins associated with mortality. In a, GO terms are grouped into biological process (BP), cellular component (CC), and molecular function (MF).


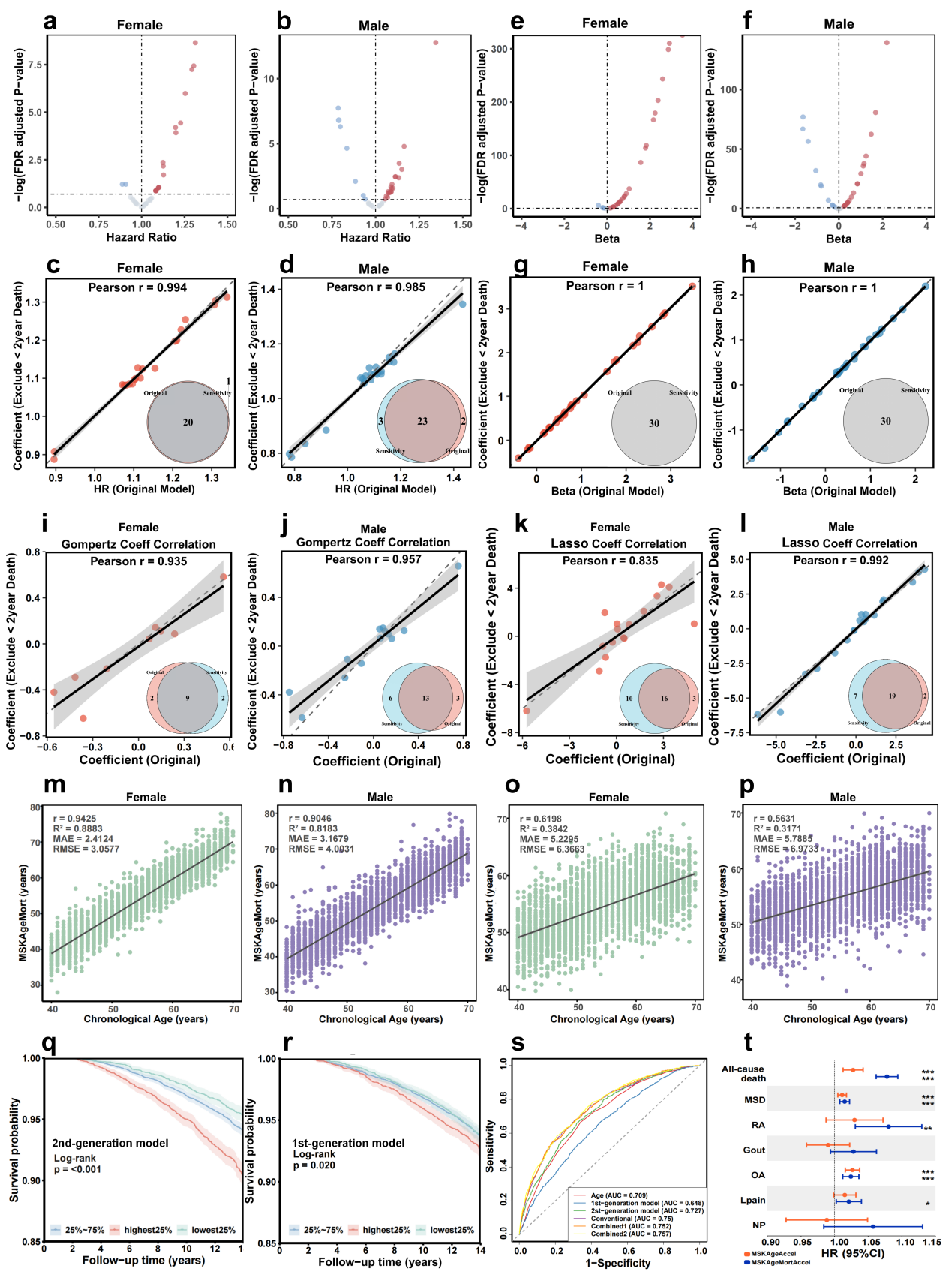


**Supplementary Figure 5.** **Sensitivity analysis of musculoskeletal aging models after excluding participants with early incident diseases or death within two years after baseline. a-b** associations between proteins and all-cause mortality in the sensitivity analysis for females and males, respectively. **c-d** concordance of hazard ratios for mortality-associated proteins between the original and sensitivity analyses. **e-f** associations between proteins and chronological age in the sensitivity analysis for females and males, respectively. **g-h** concordance of regression coefficients for chronological age-associated proteins between the original and sensitivity analyses. **i-j** correlation of model coefficients for the 2nd-generation musculoskeletal aging model, MSKAgeMort, between the original and sensitivity analyses in females and males. **k-l** correlation of model coefficients for the 1st-generation musculoskeletal aging model, MSKAge, between the original and sensitivity analyses in females and males. **c-l** with inset Venn diagrams showing the overlap of selected proteins. **m-n** correlations between predicted MSKAgeMort and chronological age in females and males. **o-p** correlations between predicted MSKAge and chronological age in females and males. **q-r** Kaplan-Meier survival curves according to aging acceleration groups derived from MSKAgeMort and MSKAge, respectively. **s** receiver operating characteristic curves comparing the predictive performance of chronological age, MSKAge, MSKAgeMort, and combined models for all-cause mortality. **t** associations of MSKAgeAccel and MSKAgeMortAccel with all-cause mortality and musculoskeletal outcomes in the sensitivity cohort. Error bars represent 95% confidence intervals. CA, chronological age; HR, hazard ratio; MAE, mean absolute error; MSD, musculoskeletal disorders; RA, rheumatoid arthritis; OA, osteoarthritis; NP, neck pain.


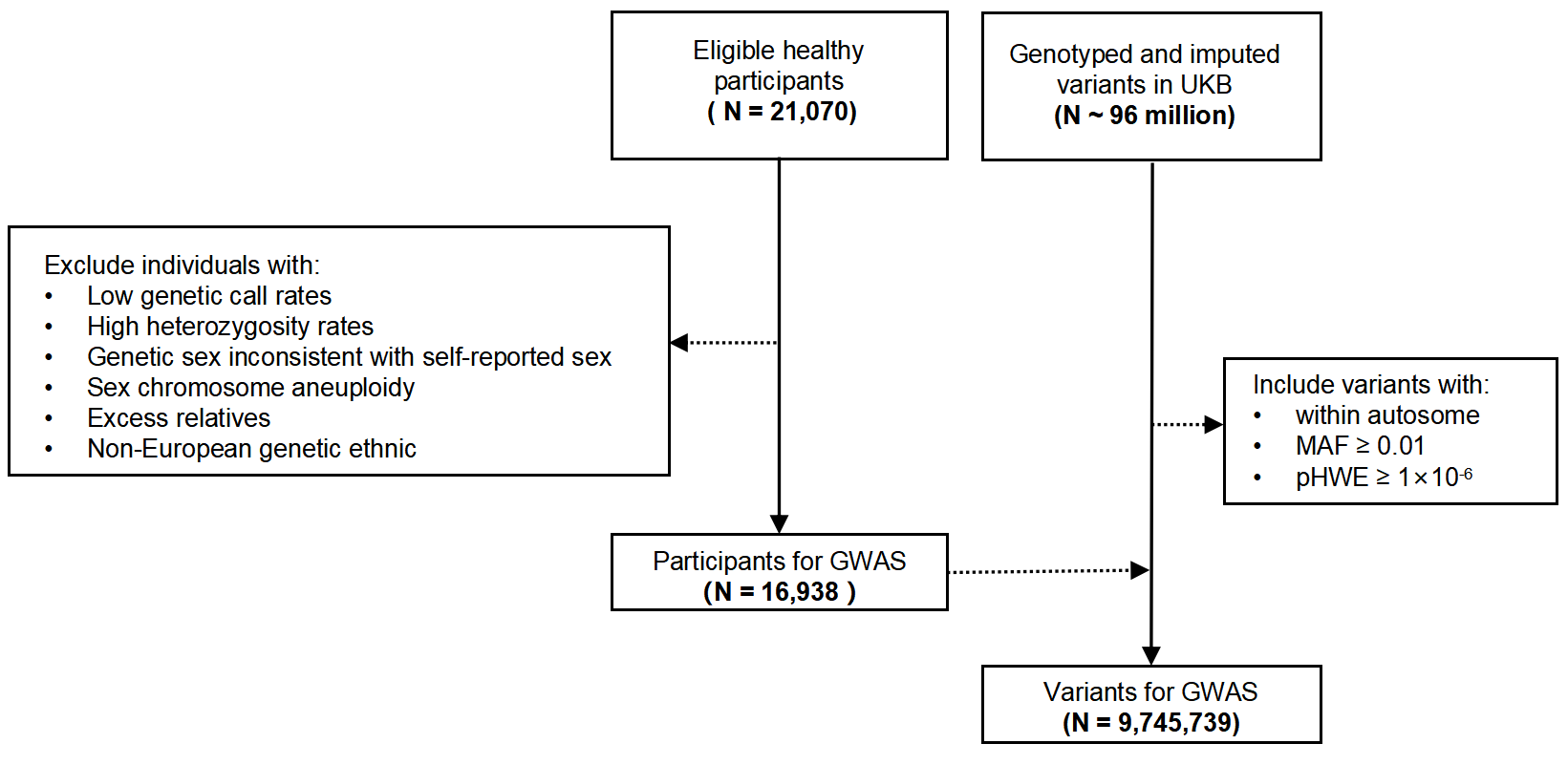


**Supplementary Figure 6.** Flowchart of GWAS quality control processes. GWAS, genome-wide association study; UKB, UK Biobank; MAF, Minor Allele Frequency; HWE, Hardy-Weinberg equilibrium.


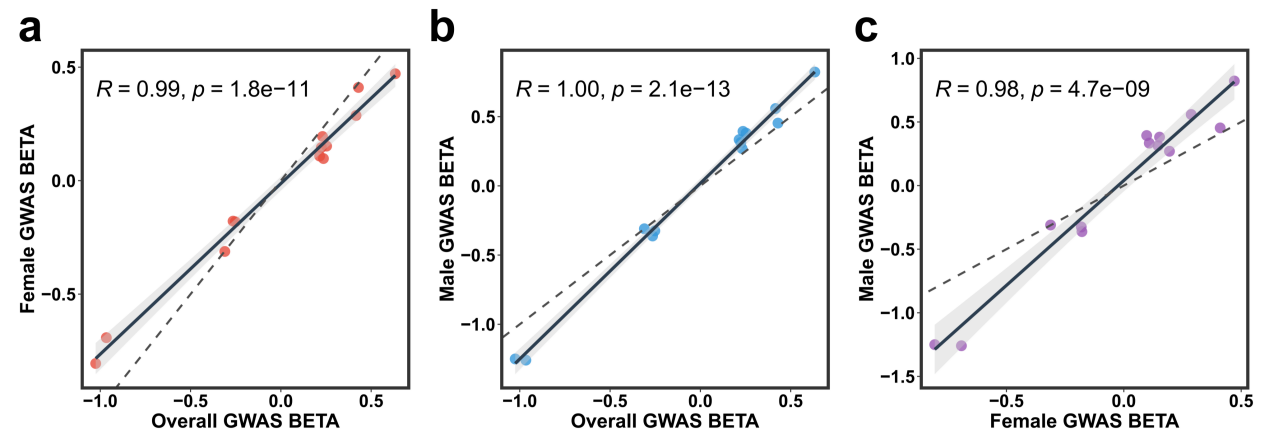


**Supplementary Figure 7. Concordance of genetic effect estimates across overall and sex-stratified GWAS of MSKAgeMortAccel.** **a** correlation of SNP effect estimates between the overall GWAS and female-stratified GWAS. **b** correlation of SNP effect estimates between the overall GWAS and male-stratified GWAS. **c** correlation of SNP effect estimates between female- and male-stratified GWAS. Points represent independent lead SNPs, the solid line represents the fitted regression line, the shaded area indicates the 95% confidence interval, and the dashed line indicates the line of equality. Pearson correlation coefficients and corresponding P values are shown in each panel.


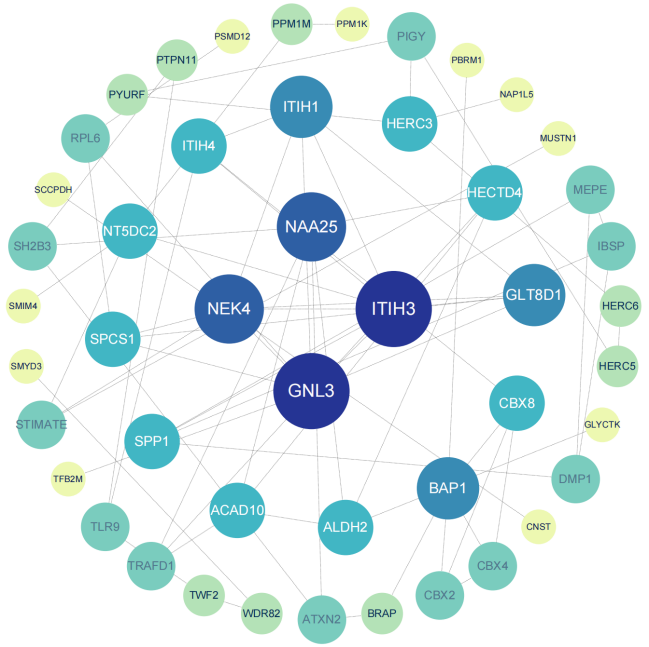


**Supplementary Figure 8. Protein–protein interaction network of candidate genes of MSKAgeMortAccel.** The circle with deeper color indicating a higher connectivity degree of gene. Dark blue represents the hub genes. MAGMA, Multi-Marker Analysis of GenoMic Annotation.
